# Establishment of a Developmental Compartment Requires Interactions between Three Synergistic *Cis*-regulatory Modules

**DOI:** 10.1371/journal.pgen.1005376

**Published:** 2015-10-15

**Authors:** Dimitri Bieli, Oguz Kanca, David Requena, Fisun Hamaratoglu, Daryl Gohl, Paul Schedl, Markus Affolter, Matthew Slattery, Martin Müller, Carlos Estella

**Affiliations:** 1 Biozentrum, University of Basel, Basel, Switzerland; 2 Departamento de Biología Molecular and Centro de Biología Molecular Severo Ochoa, Universidad Autónoma de Madrid (UAM), Madrid, Spain; 3 Center for Integrative Genomics, University of Lausanne, Lausanne, Switzerland; 4 Department of Molecular Biology, Princeton University, Princeton, New Jersey, United States of America; 5 Department of Biomedical Sciences, University of Minnesota Medical School. Duluth, Minnesota, United States of America; New York University, UNITED STATES

## Abstract

The subdivision of cell populations in compartments is a key event during animal development. In *Drosophila*, the gene *apterous (ap)* divides the wing imaginal disc in dorsal *vs* ventral cell lineages and is required for wing formation. *ap* function as a dorsal selector gene has been extensively studied. However, the regulation of its expression during wing development is poorly understood. In this study, we analyzed *ap* transcriptional regulation at the endogenous locus and identified three *cis*-regulatory modules (CRMs) essential for wing development. Only when the three CRMs are combined, robust *ap* expression is obtained. In addition, we genetically and molecularly analyzed the trans-factors that regulate these CRMs. Our results propose a three-step mechanism for the cell lineage compartment expression of *ap* that includes initial activation, positive autoregulation and Trithorax-mediated maintenance through separable CRMs.

## Author Summary

The separation of cell populations into distinct functional units is essential for both vertebrate and invertebrate animal development. A classical paradigm for this phenomenon is the establishment of developmental compartments during *Drosophila* wing development. These compartments depend on the restricted expression of two selector genes, *engrailed* in the posterior compartment and *apterous* (*ap*) in the dorsal compartment. Yet, despite the central role these genes and their restricted expression patterns play in *Drosophila* development, we still do not understand how these patterns are established or maintained. Here, by dissecting the regulatory sequences required for *ap* expression, we solve this problem for this critical selector gene. We used a combination of experimental approaches to identify and functionally characterize the *cis*-regulatory modules (CRMs) that regulate *ap* expression during *Drosophila* wing development. For these analyses we implement a novel technique allowing us to study the function of these CRMs *in vivo*, at the native *ap* locus. We found three *ap* CRMs crucial for wing development: the Early (apE) and the D/V (apDV) enhancers and the *ap* PRE (apP). Only when all three regulatory elements are combined is a uniform and complete *ap* expression domain generated. In summary, our results indicate that *ap* is regulated in time and space by a three-step mechanism that generates a lineage compartment by integrating input from separate CRMs for the initiation, refinement and maintenance of its expression.

## Introduction

Animal development requires the segregation of cell populations using both lineage and non-lineage boundaries. These cell boundaries act as signaling centers that organize the growth and patterning of specific tissues (reviewed in [1]). A paradigmatic example is the subdivision of the *Drosophila* wing disc into anterior-posterior (A/P) and dorsal-ventral (D/V) compartments. At the compartment boundaries, ligands encoded by *decapentaplegic* (*dpp*) and *wingless* (*wg*) are secreted and activate signaling pathways that orchestrate wing development [2–8]. The generation of the A/P and D/V compartments is directed by specific transcription factors, the selector genes *engrailed* (*en*) and *apterous* (*ap*), respectively, that define the identity of the cells using a binary code (on or off) [9–15]. Once the compartmental fates have been assigned, the cells in which *en* and *ap* are expressed as well as their descendants maintain that “determined” state. Unlike the A/P wing division, which is established during embryonic development, the D/V boundary is defined in the wing disc during the second larval stage by the expression of *ap* [16]. *ap* encodes a LIM-type homeodomain transcription factor and its activity depends on the formation of a complex with the LIM-domain binding protein Chip [17–19]. Since *ap* function is crucial to initiate the signaling center at the D/V boundary [20,21], *ap* null mutants completely lack the wing [16].

Due to its key role in wing disc development, *ap* function has been studied extensively. However, the transcriptional regulation of *ap* is poorly understood. How a sharp border of *ap*-expressing and non-expressing cells is generated *de novo* during the growth phase of the imaginal disc, and how the expression of *ap* is maintained and restricted to the dorsal compartment are critical unanswered aspects of wing development.

The spatial and temporal regulation of gene expression is mediated by the binding of transcription factors to discrete DNA sequences named *cis*-regulatory modules (CRMs). CRMs can be located up to hundreds of kilobases away from their target promoters. Synergistic interactions between CRMs may be required to faithfully regulate gene transcription (reviewed in [22]). Several CRMs have been identified controlling *ap* expression in different tissues, such as in muscle progenitors and in the embryonic nervous system [23,24]. A wing disc specific enhancer, named apC, has been reported to drive expression in the dorsal wing disc [24]. However, it has been demonstrated that this element is not sufficient for proper *ap* regulation in the wing [25]. *ap* expression is initially activated in future dorsal cells by the *Drosophila* Epidermal Growth Factor Receptor (EGFR) pathway through the secreted neuregulin-like signaling protein Vein (Vn) [26,27]. However, it is still unknown how *ap* expression is regulated after this initial EGFR-mediated activation. This is particularly critical in a highly proliferating tissue such as the wing imaginal disc.

The maintenance of selector gene expression domains through multiple rounds of cell divisions partially depends on the activity of the Polycomb and Trithorax group gene products (PcG and TrxG). These proteins either repress (PcG) or activate (TrxG) the expression of their target genes through *cis*-regulatory sequences called Polycomb Response Elements (PREs) (reviewed in [28,29]). It has been suggested that *ap* expression is repressed by PcG protein complexes in ventral wing disc cells [30].

In this study, we have analyzed the regulation of *ap* at the endogenous locus and identified three *ap* CRMs crucial for wing development: the Early (apE) and the D/V (apDV) enhancers and the *ap* PRE (apP). Importantly, we analyzed these CRMs in the endogenous locus using a novel *in situ* rescue system. We find that only when the three regulatory elements are combined, a uniform and complete *ap* expression domain is observed. Our results indicate that *ap* is regulated by a three-step mechanism that generates a lineage compartment through the integration of input from separate CRMs for the initiation, refinement and maintenance of its expression.

## Results

### Genetic characterization of the *apterous* promoter region

**Fig 1:**
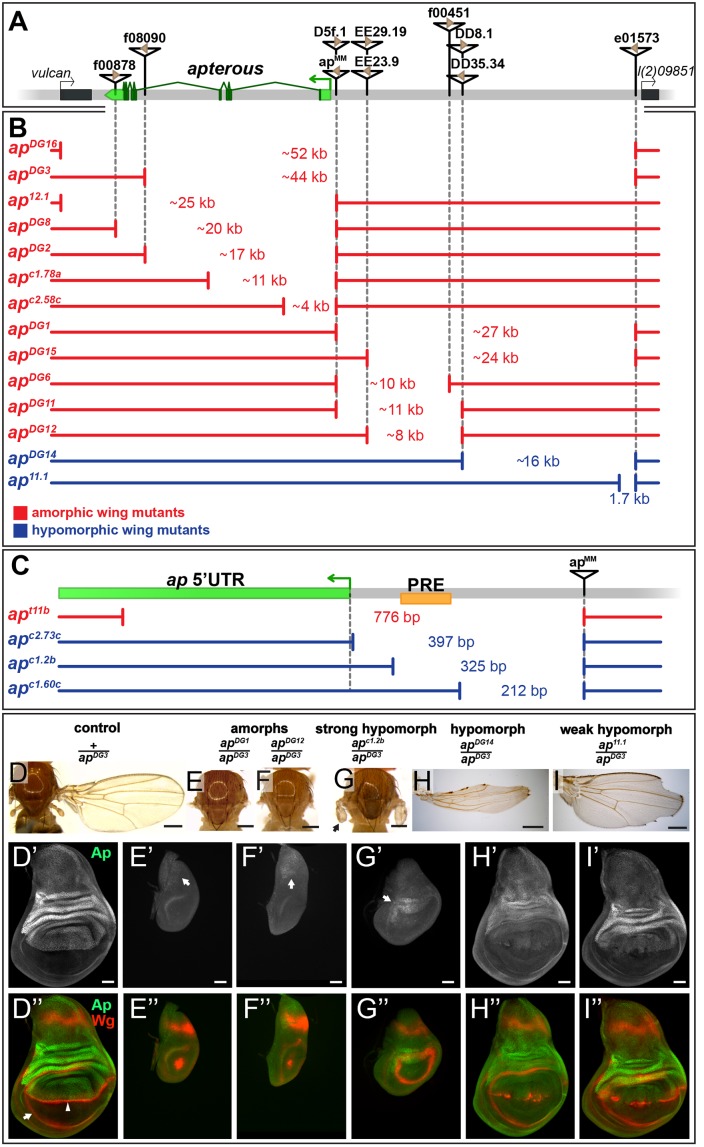
Deletion analysis at the *ap* locus. **(A)** Overview of the *ap* gene locus. *ap* transcript *ap-RA* is indicated in green and the arrow at the 5’ end demarcates its TSS. The flanking genes (indicated by black boxes) are *vulcan* on the proximal and *l(2)09851* on the distal side. Relevant transposable elements used for the generation of deletions by *flp*-mediated recombination are displayed as black triangles with FRT sites within them as brown triangles. FRT orientation is indicated as defined by [82]. **(B and C)** Deletions at the *ap* locus. The number in between the break points indicates the approximate length of the deletion. Phenotypically, the deletions can be divided into amorphic (in red) or hypomorphic (in blue) wing alleles when hemizygous over *ap* ^*DG3*^. Please note the different scales of the maps depicted in B and C. **(B)** Deletions that affect the coding sequence all lead to a no wing phenotype (*ap* ^*DG16*^, *ap* ^*DG3*^, *ap* ^*12*.*1*^, *ap* ^*DG8*^, *ap* ^*DG2*^, *ap* ^*c1*.*78a*^, and *ap* ^*c2*.*58c*^). Deletions in the upstream noncoding region between *ap* ^*MM*^ and *l(2)09851* either lead to amorphic (*ap* ^*DG1*^, *ap* ^*DG15*^, *ap* ^*DG6*^, *ap* ^*DG11*^, and *ap* ^*DG12*^) or hypomorphic wing phenotypes (*ap* ^*DG14*^ and *ap* ^*11*.*1*^). **(C)** Blow up of the *ap* promoter region specific for transcripts *ap-RA* and *ap-RC*. The PRE core is depicted by a yellow box. *ap* ^*t11b*^, a deletion which removes the TSS as well as the PRE core, results in a no wing phenotype. The two deletions *ap* ^*c2*.*73c*^ and *ap* ^*c1*.*2b*^ leave the TSS intact but both remove the PRE core and both yield strong hypomorphic wing phenotypes. The weak hypomorphic allele *ap* ^*c1*.*60c*^ leaves TSS and PRE core untouched. **(D-I)** Wings and 3^rd^ instar wing discs of representative *ap* wing mutants stained for Wg (red) and Ap (green). **(D)** Wing and notum of a hemizygous +/*ap* ^*DG3*^ fly. Almost 100% of the wings look normal [25] **(D’)** Ap staining in the wing disc demarcates the dorsal compartment. **(D”)** Wg staining: the inner ring outlines the wing pouch (white arrow) and the stripe traversing it corresponds to the D/V compartment boundary (white arrowhead). **(E and F)** All wing tissue is lost in amophic wing mutants (*ap* ^*DG1*^/*ap* ^*DG3*^ and *ap* ^*DG12*^/*ap* ^*DG3*^). **(E’ and F’)** Only weak Ap staining is detectable in the notum (white arrow). **(E” and F”)** The wing pouch is completely lost and the inner Wg ring is reduced to a dot. **(G)** In strong hypomorphic conditions (*ap* ^*c1*.*2b*^/*ap* ^*DG3*^), only small wing and haltere stumps form (black arrow). **(G’)** Low Ap protein levels are detected (white arrow) mainly in the hinge region. **(G”)** The size of the wing pouch is drastically reduced and no Wg stripe along the D/V boundary is visible. **(H)** Hypomorphic mutants (*ap* ^*DG14*^/*ap* ^*DG3*^) developed considerably more wing tissue with no or little wing margin or hinge. **(H’)** Compared to control discs, weaker Ap staining is observed in the pouch region. **(H”)** The size of the wing pouch is comparable to wild type while the D/V Wg stripe is disrupted. **(I)** In weak hypomorphic mutants (*ap* ^*11*.*1*^/*ap* ^*DG3*^) notching of the wing blade is prominent. **(I’)** Compared to control discs, *ap* expression is mostly compromised in the pouch region. **(I”)** Pouch size is similar to wild type and the Wg D/V stripe is locally disrupted. All scale bars are 50 μm.

*ap* is expressed in multiple tissues during embryonic and larval stages. Four different transcripts starting from three different promoters have been annotated which give rise to three unique polypeptides (FlyBase). We have generated a series of deletions to identify which *ap* non-coding sequences are required for *ap* expression in the wing imaginal disc (Fig 1A–1C; see Materials and Methods for information about each allele). Unless otherwise stated, hemizygous phenotypes resulting from these deletions were analyzed over *ap* ^*DG3*^, a large deletion removing the bulk of the *ap* locus [25], and were classified as amorphs or hypomorphs depending on their severity. *ap* amorphs were defined by the absence of wing tissue in discs and adults.

The shortest deletion in our collection with an *ap* null phenotype is *ap* ^*t11b*^ (Fig 1C and S1D Fig). It specifically deletes the transcription start site (TSS) of transcripts *ap-RA* and *ap-RC*. Our *in silico* analysis indicates that this TSS is not controlled by a TATA-box promoter, but rather contains an Initiator (Inr) and a Downstream Promoter Element (DPE) (for review see [31]). In addition, *ap* ^*t11b*^ removes a PRE located around 100 bp upstream of the *ap-RA/ap-RC* TSS. This PRE was defined by several chromatin immunoprecipitation studies with various anti-PcG antibodies [30,32–34]. The putative PRE core as defined by Oktaba *et al* [30] is indicated in Fig 1C. Two other small deletions with the same distal break point as *ap* ^*t11b*^ were isolated: *ap* ^*c2*.*73c*^ and *ap* ^*c1*.*2b*^. They leave the TSS, Inr and DPE intact, but remove the PRE core. In hemizygous flies, small wing stumps are often formed (Fig 1G and S1E Fig). In addition the wing pouches of 3^rd^ instar wing discs are larger than in amorphic mutants (compare Fig 1G” with 1F”). Small amounts of Ap can only be detected in the presumptive hinge and notum (arrows in Fig 1G’ and S1E Fig). The Wg stripe along the compartment boundary is absent (Fig 1G” and S1E Fig). Hence, *ap* ^*c2*.*73c*^ and *ap* ^*c1*.*2b*^ behave as strong hypomorphic alleles. A dramatic improvement of the adult wing phenotype is observed for deletion *ap* ^*c1*.*60c*^ which is a mere 113 bp shorter than *ap* ^*c1*.*2b*^ (Fig 1C and S1F Fig). Note that it keeps the TSS, Inr, DPE as well as the PRE core in place. A weak phenotype becomes apparent in hemizygous condition: similar to other weak *ap* loss-of-function alleles, most wing margins have notches. Unexpectedly, this phenotype is brought about by partial ectopic *ap* expression in the ventral pouch compartment which correlates with gaps in the Wg stripe along the compartment boundary (S1F Fig).

In summary, these observations provide strong genetic evidence for an important contribution of the *ap* PRE to wing development. In addition, a region defined by *ap* ^*c1*.*60c*^ appears to act as an auxiliary module, which helps to confine the established Ap pattern to the dorsal compartment.

### Deletions affecting the intergenic spacer separating *ap* and *l(2)09851* identify two regions important for *ap* function

As a next step, we generated alleles that retain an intact PRE/promoter region, but remove upstream non-coding regions of *ap*. In the mutant *ap* ^*DG1*^, 27 kb of the upstream non-coding region are deleted (Fig 1B) [35]. Hemizygous flies of this genotype can be considered as amorphic mutants, since no wing tissue was formed despite weak residual *ap* expression in the notum (Fig 1E’). Removing proximal upstream regions (*ap* ^*DG6*^, *ap* ^*DG11*^, and *ap* ^*DG12*^) also resulted in amorphic phenotypes (Fig 1B and 1F). These deletions remove the previously identified *ap* wing enhancer apC [24,25]. The distal part of the interval defined by *ap* ^*DG1*^ was deleted in *ap* ^*DG14*^. Hemizygous flies form wing stumps of almost normal length but wing margin formation is severely impaired (Fig 1H). In the corresponding wing discs, *ap* expression in the wing pouch is reduced and the Wg stripe along the D/V boundary is critically perturbed (Fig 1H’ and 1H”). The large size of the a*p* ^*DG14*^ deficiency precludes a precise localization of an enhancer element within the ~16 kb interval. However, a few small deletions extending only proximal to e01573 allowed us to narrow down its approximate distal end: one of them, *ap* ^*11*.*1*^, deletes 1654 bp (Fig 1B; see also Materials and Methods). It can be maintained as a homozygous stock and wings look wild-type. Its weak hypomorphic nature is revealed in hemizygous *ap* ^*11*.*1*^ flies: all wings have notches along the margin (Fig 1I). Their origin can be traced to gaps in the Wg stripe along the D/V compartment boundary due to reduced Ap levels in the pouch (Fig 1I’ and 1I”).

### Two conserved regions harbor essential wing enhancer elements

**Fig 2:**
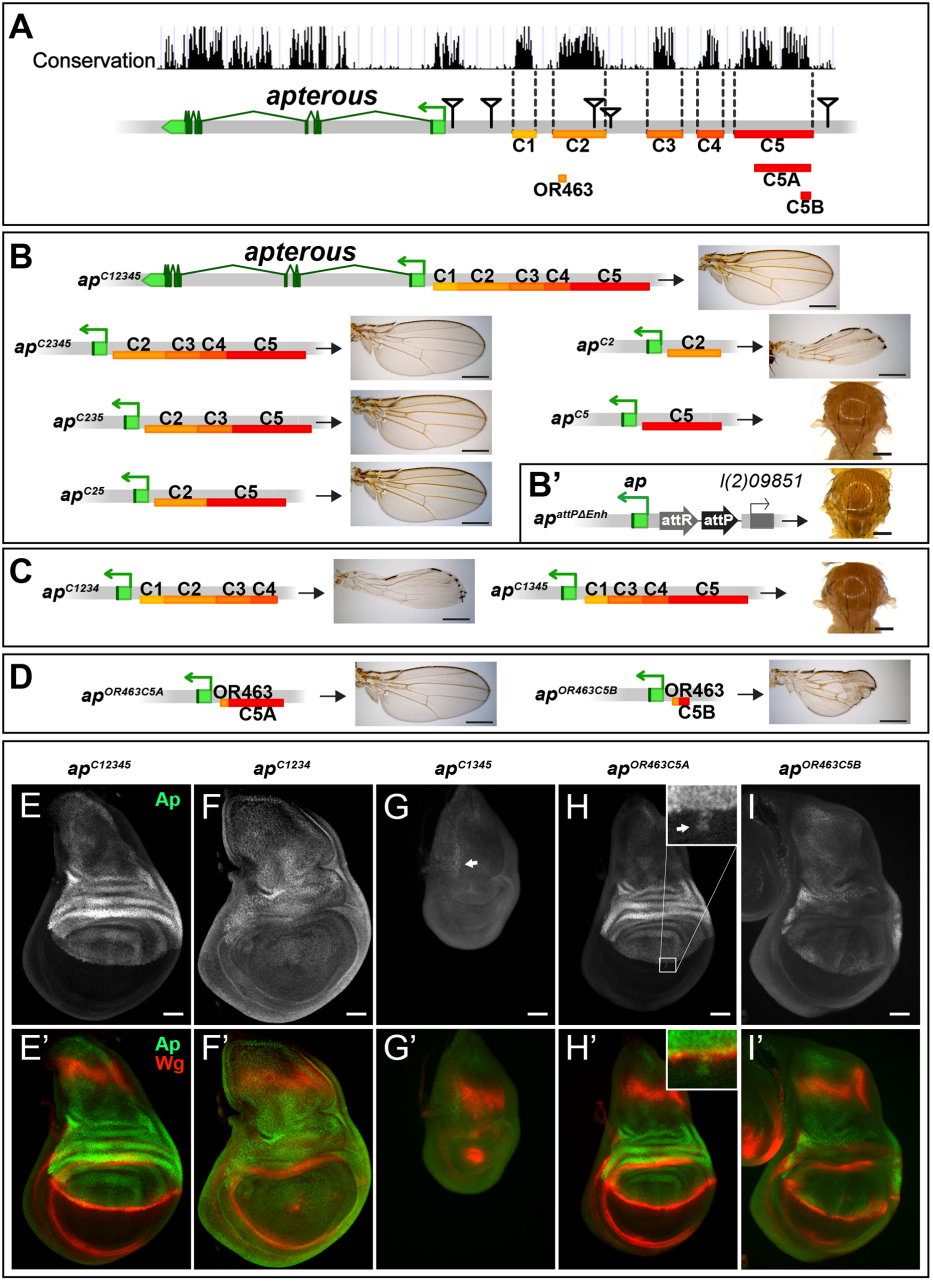
Analysis of the *ap* wing enhancer region. **(A)** Conservation of the *ap* locus (data from UCSC genome browser) and subdivision of the 27 kb intergenic region between *ap* and *l(2)09851* into 5 conserved blocks (C1–C5) is shown. OR463, C5A and C5B are subfragments of C2 and C5, respectively. Black triangles mark the locations of the transposon used for the generation of the deletions. **(B)** Six different constructs consisting of variable combinations of conserved blocks and the corresponding hemizygous wing phenotypes are depicted. When all 5 conserved regions are present (*ap* ^*C12345*^), a normal sized and patterned wing develops. Gradual removal of C1 (*ap* ^*C2345*^), C4 (*ap* ^*C235*^), and C3 (*ap* ^*C25*^) has no effect on wing morphology. Removing C5 from *ap* ^*C25*^ results in hypomorphic wings (*ap* ^*C2*^). C5 alone (*ap* ^*C5*^) is an amophic allele, as no wings are formed. **(B’)** *ap* ^*attPΔEnh*^: the docking site of the *in situ* rescue system for the evaluation of DNA fragments originating from the 27 kb intergenic spacer is shown. An attP site located ~400 bp upstream of the *ap* TSS juxtaposes the promoter/PRE region. As in *ap* ^*DG1*^, the 27 kb intergenic region is deleted. **(C)** Removing C2 or C5 in the context of *ap* ^*C12345*^ (*ap* ^*C1234*^ and *ap* ^*C1345*^) leads to the same phenotype as each element alone (*ap* ^*C2*^ or *ap* ^*C5*^, respectively). **(D)** Enhancer bashing of C2 and C5 regions. OR463 and C5A in combination are the shortest fragments that still result in a normal wing (*ap* ^*OR463C5A*^). C5B, a sub-fragment of C5A, in combination with OR463 does not fully rescue wing formation (*ap* ^*OR463C5B*^). Wing size is reduced, but all margin structures are formed. **(E-I)** Third instar wing discs of different genotypes stained for Ap (green) and Wg (red). **(E-E’)** *ap* ^*C12345*^: Ap and Wg pattern is indistinguishable from wild type. **(F-F’)** *ap* ^*C1234*^: a significant reduction of Ap levels in the wing pouch is observed. The Wg stripe along the D/V border is almost completely lost. **(G-G’)** *ap* ^*C1345*^: scattered cells with little Ap protein are detectable in the notum (see arrow). Wing pouch is reduced to a small dot of *wg* expression. **(H-H’)** *ap* ^*OR463C5A*^: Ap and Wg patterns are similar to wild type. Ap protein can sometimes be detected in some cells of the ventral part of the disc (arrow in inset). **(I-I’)** *ap* ^*OR463C5B*^: although protein levels are reduced, the Ap pattern is close to wild type. Nevertheless, the appearance of the Wg stripe along the D/V border is not as smooth as in wild type. All scale bars are 50 μm.

To further characterize the intergenic spacer between *ap* and *l(2)09851*, we engineered and validated a system which allowed us to investigate the role of given DNA stretches at the *ap* locus [25]. Briefly, we deleted the 27 kb (*ap* ^*DG1*^) upstream region of *ap*, and replaced it by an attP site juxtaposing the promoter/PRE (*ap* ^*attPΔEnh*^; Fig 2B’). In this amorphic situation, we were able to bring back sub-fragments of the previously deleted regulatory regions by ΦC31-integrase mediated insertion and test their ability to rescue wing development. Again, all the newly generated alleles were tested in hemizygous condition. According to sequence conservation and histone H3 lysine 4 trimethylation (H3K4me3) patterns, which have been reported to correlate with active promoters and enhancers [36], we have divided the upstream non-coding region of *ap* into 5 blocks (C1–5, Fig 2A). Combining all 5 conserved blocks and reintroducing them into *ap* ^*attPΔEnh*^ (*ap* ^*C12345*^), fully rescued wing formation (Fig 2B) as well as the Ap and Wg pattern in wing imaginal discs (compare Figs 2E with 1D). Deleting the conserved blocks that showed no H3K4me3 mark (C1 and C4), had no consequence on wing phenotype (Fig 2B). Next, we deleted conserved regions with a methylation mark. Deleting C3 had no influence on wing formation (*ap* ^*C25*^, Fig 2B). In contrast, upon removal of C5, wing development was critically disturbed (*ap* ^*C2*^, Fig 2B). Long wing stumps with defective wing margin and hinge were formed that resembled the hypomorphic *ap* ^*DG14*^ mutant, which completely lacks the conserved C5 block (compare Figs 2B and 1H). In flies containing only C5, no wing tissue was formed (*ap* ^*C5*^).

We then tested whether the C2 and C5 fragments were also necessary when all the other conserved elements were present. Removing C5 only (*ap* ^*C1234*^) had the same effect as maintaining C2 alone, since long wing stumps with little margin and hinge were formed (compare Fig 2C with 2B). Wing discs of this genotype showed drastically reduced *ap* expression in the pouch region and in most cases lost the Wg stripe along the D/V boundary (Fig 2F). As expected, when only C2 was removed (*ap* ^*C1345*^, see Fig 2C), no wing tissue was formed, Ap protein was only weakly detected in the notum and the Wg pattern was equivalent to amophic *ap* wing mutants (Fig 2G).

We also investigated a possible role of the positions of C2 and C5 relative to each other in *ap* ^*C52*^ and *ap* ^*C15342*^ flies. Both alleles yield wild-type wings in hemizygous flies, indicating that their order on the chromosome is not important (S2A Fig).

Next, we aimed at defining the minimal CRMs which were able to direct wing development. We have recently found that shorter sub-fragments of C2 retain its wing disc specific activity [25]. The combination of a 463 bp fragment of C2 (OR463) and 3.8 kb of C5 (C5A) in *ap* ^*OR463C5A*^ fully rescued wing development (Fig 2D and 2H). Replacing C5A by C5B, a 600 bp subfragment of C5A, indicated that it lacks certain regulatory input (Fig 2D). The expression of *ap* in *ap* ^*OR463C5B*^ wing discs was restricted to the dorsal compartment, but reduced compared to *ap* ^*C12345*^ (compare Fig 2I with 2E). Nevertheless, apart from small disruptions at the D/V boundary, *wg* expression appeared almost normal (Fig 2I’).

Finally, to investigate whether additional wing-specific CRMs reside within the intronic sequences, we replaced the coding sequences with an *ap* cDNA lacking most intronic sequences (*ap* ^*cDNAint2*.*3*^). This allele produces normal wings (S2B and S2C Fig). Thus, we conclude that no essential wing CRMs are present in the intronic regions of *ap*. In agreement with this notion, fragments taken from intronic sequences (see below and S2D Fig) failed to drive reporter gene expression in the wing disc. Note that the cDNA used for the construction of *ap* ^*cDNAint2*.*3*^ corresponds to the *ap-RA*/*ap-RC* transcripts.

Combining the results from the two complementary *in vivo* approaches (deletion analysis and the *in situ* rescue system), we have defined three distinct regions which are absolutely required for the correct *ap* expression in the wing disc: a region next to the *ap* TSS which contains a PRE and two enhancers with distinct regulatory input located in homology blocks C2 and C5.

### Identification of *ap cis*-regulatory modules active in the wing imaginal disc

**Fig 3:**
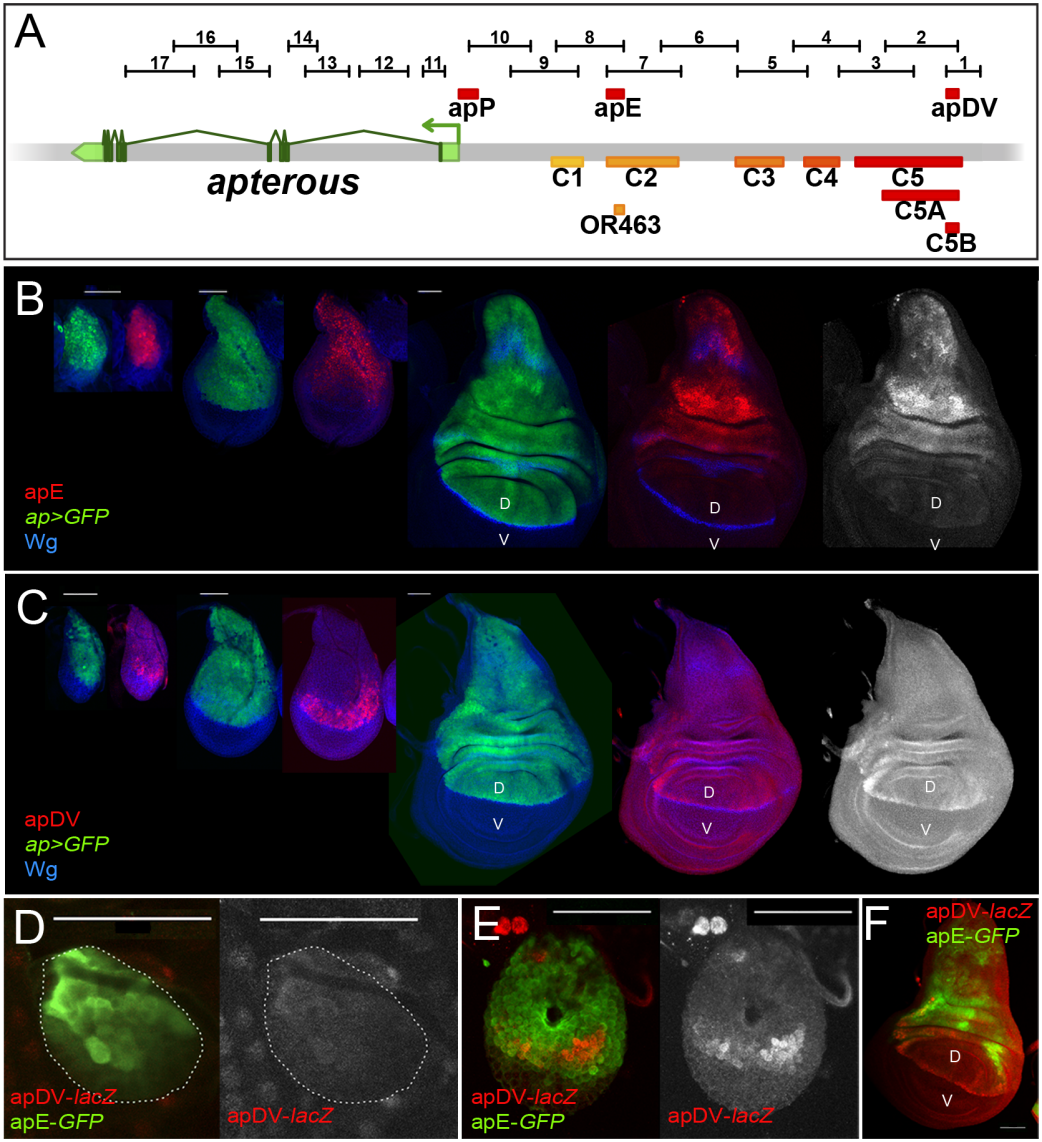
Activity patterns of apE and apDV enhancers. **(A)** Schematic representation of the *ap* genomic region is shown as a grey bar. *ap* transcript *ap-RA* is shown in green. In the upper part of the panel, horizontal bars represent the DNA elements for which Gal4 drivers were generated by the Janelia Farm consortium except for line 6 (see Materials and Methods) (http://flweb.janelia.org/cgi-bin/flew.cgi). Red bars represent regulatory elements apP, apE and apDV. At the bottom of the panel, 8 fragments tested with the *ap in situ* rescue system are indicated. **(B-C)** Pairs of wing imaginal discs isolated from second, early and late third instar larvae are shown (from left to right). They were stained for GFP (*ap*-*Gal4*>UAS-*GFP*, green) and Wg (blue) or apE-*lacZ* (red) in (B) or apDV-*lacZ* (red) in (C). Note that *ap>GFP* represents the complete *ap* pattern to which those of apE-*lacZ* and apDV-*lacZ* are compared. **(B)** In early discs, apE is active in all the cells that express *ap*. Later, its activity is restricted to a subset of *ap*-expressing cells mainly in the notum and hinge region. Expression in the wing pouch is very low. **(C)** apDV is active in dorsal-distal cells in early discs. Later, its activity is restricted to dorsal wing pouch cells close to the D/V boundary. **(D-F)** Early second **(D),** mid-second **(E)** and third instar imaginal discs **(F)** stained for apDV-*lacZ* (red) and apE-*GFP* (green) are shown. **(D)** apE is activated earlier than apDV in proximal wing disc cells. **(E)** apDV is activated in dorsal-distal cells that already have apE activity. **(F)** In third instar imaginal discs, apE and apDV occupy complementary territories. apDV is restricted to dorsal wing cells close to the D/V border. apE remains mainly active in the hinge and notum. All scale bars are 50 μm. D, dorsal and V, ventral.

In parallel to the *ap* deletion and *in situ* rescue strategies, we performed an unbiased search for *ap* CRMs active in the wing imaginal disc. Using the Fly Light database [37] and self-made constructs (see Materials and Methods), we screened the *ap* genomic region for DNA fragments that activate the *Gal4* gene in an *ap*-like expression pattern (Fig 3A). We found that 4 of the 17 lines tested partially recapitulated *ap*-like expression pattern in third instar wing imaginal disc (S3 Fig). Interestingly, lines 1 and 2 were active in a similar pattern in the wing pouch and hinge but were not active in the notum, while lines 7 and 8 showed identical expression pattern in the notum and hinge with low levels in the dorsal wing pouch (S3 Fig). Subsequently, we cloned the overlapping sequences between lines 1–2 and 7–8 in reporter constructs and compared their activity with *ap* expression as well as with each other during wing imaginal disc development (Fig 3B–3F; see Materials and Methods). apE (Early), the first element to be activated in early to mid-second instar imaginal discs, drove expression in all *ap*-expressing cells (Fig 3B and 3D). The other element, named apDV (Dorso-Ventral), was activated a few hours later in dorsal cells close to the D/V boundary (Fig 3C and 3E). As the wing imaginal disc developed, the activity of apE became mainly restricted to the notum and hinge with low expression remaining in the wing pouch (Fig 3B). In contrast, apDV was always restricted to dorsal wing pouch cells close to the D/V boundary, with some cells expressing the reporter in the dorsal wing hinge (Fig 3C).

In line with our previous results, apE and apDV are located within the C2 and C5 regulatory fragments identified with the *in situ* rescue system, respectively, and overlap with OR463 and C5B (Fig 3A). Moreover, in a reporter gene construct, C2 and C5 reproduced the same expression pattern described for apE and apDV, respectively (S3A Fig).

It should be noted that none of the single *ap*-CRMs identified, apDV or apE, nor the combination of them, apDV-*lacZ*+apE-*GFP*, was able to completely reproduce the endogenous *ap* expression pattern, suggesting that additional elements are necessary (Fig 3F and see below).

### The EGFR pathway transiently regulates the apE element

**Fig 4:**
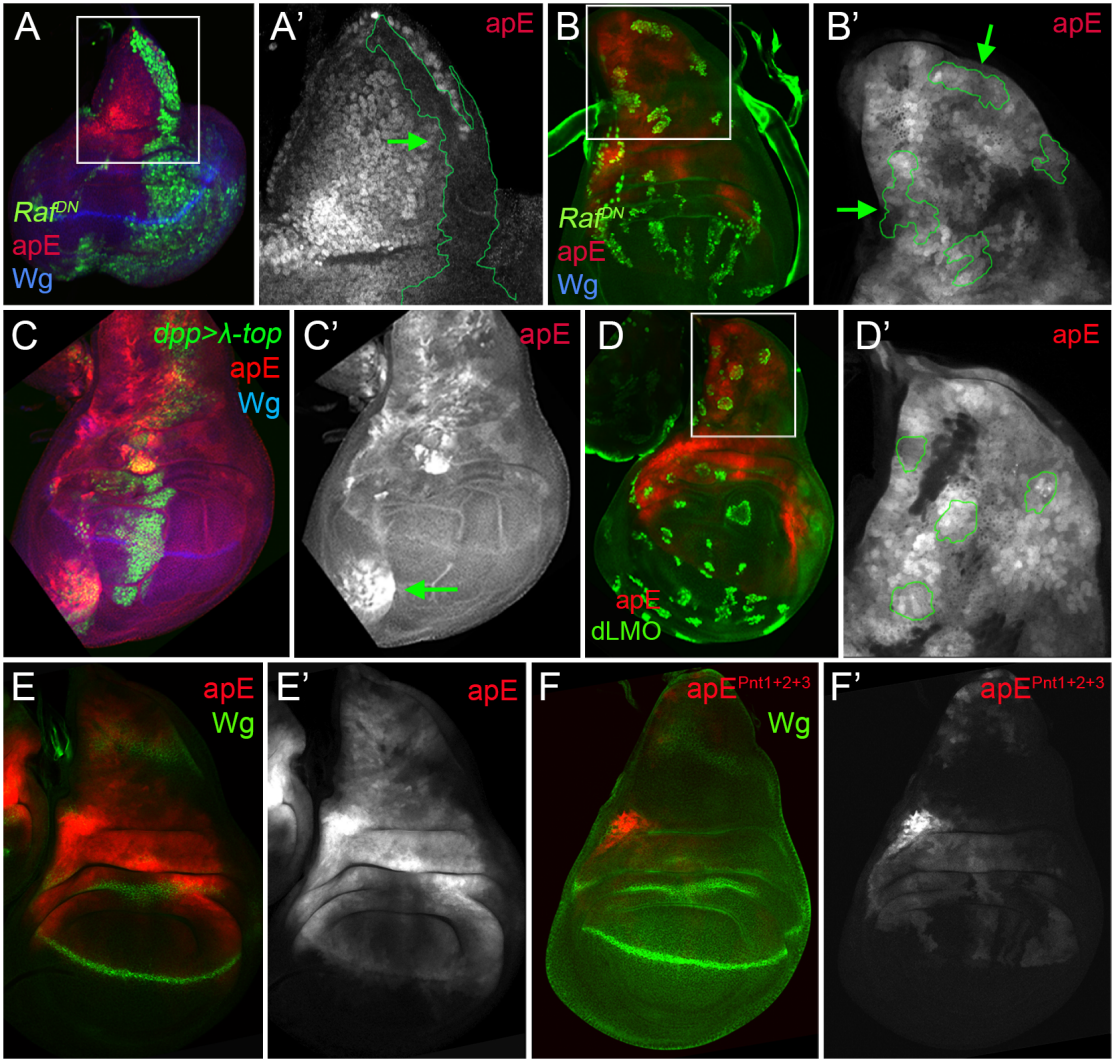
apE is regulated by the EGFR pathway. **(A-B)** Third instar wing imaginal disc with clones expressing a dominant negative version of Raf (*Raf* ^*DN*^) induced at different time points of larval development are marked by GFP (green). Discs were also stained for Wg (blue) and apE-*lacZ* activity (red). **(A)** Early induced *Raf* ^*DN*^ clones (24–48hrs after egg laying, AEL) are unable to activate apE. **(A´)** Close-up of the disc in **(A)** with the clone outlined in green (green arrow). Note that apE is not activated within the clone. **(B)** Late induced *Raf* ^*DN*^ clones (72–96hrs AEL) have no effect on apE activity. **(B´)** Close-up of the disc in **(B)** with clones outlined in green (green arrows). **(C)** *dpp*-*Gal4*; UAS-*GFP*, UAS-*EGFR* ^*λtop4*.*2*^ (*λ-top*, green) wing disc stained for apE-*lacZ* (red) and Wg (blue). Ectopic activation of the EGFR pathway induces apE in ventral pleural cells. **(C´)** Single channel for apE. Green arrow points to ectopic apE activity. **(D)** Gain of function clones of the Ap activity repressor dLMO (green) has no effect on apE activity (red). **(D´)** Close-up of the disc in **(D)** with clones outlined in green. **(E-F)** Wing imaginal discs stained for Wg (green) and apE (**E**, red) and apE^pnt1+2+3^ (**F**, red) activity. Note that apE activity is strongly reduced after mutating the three identified Pnt binding sites (apE^pnt1+2+3^). All constructs have been inserted in the same genomic location. Images were obtained keeping the confocal settings constant.

The initial *ap* expression in the wing disc is activated by the EGFR signalling pathway at early stages of wing development (from early to mid-second instar), while its later expression is EGFR-independent [26,27]. Since the apE element was active in the entire *ap* expression domain in early wing discs, we tested whether this CRM is regulated by the EGFR pathway. Clones of cells expressing a dominant-negative form of the pathway effector Raf (*Raf* ^*DN*^) generated early in larval development (24–48 hrs after egg laying, AEL) were unable to activate apE (Fig 4A), while no effect was observed in clones generated later (72–96h AEL, Fig 4B). The same temporal EGFR-dependency of apE was found when the pathway was reduced in the entire wing disc using a temperature-sensitive EGFR allele (S4 Fig).

Consistent with the low levels of apE activity in dorsal wing pouch cells, misexpression of a constitutive active version of the EGFR receptor (*EGFR* ^*λtop4*.*2*^) by *dpp-Gal4* activated apE-*lacZ* expression in cells of the ventral pleura, while wing pouch cells were resilient to activate it (Fig 4C). To rule out a potential auto-regulatory input on the apE element, gain of function clones of the Ap activity repressor dLMO were made [38]. However, dLMO expression had no effect on apE activity (Fig 4D). Taken together, these results suggest that apE is activated by the EGFR pathway and that other factors regulate its expression afterwards.

To understand how the EGFR pathway regulates apE activity, we searched for putative binding sites of the ETS transcription factor Pnt [39]. Two highly conserved and one less conserved sites were identified. When all these sites were mutated simultaneously, the activity of apE was strongly reduced (compare Fig 4E with 4F). Altogether, these results suggest that apE is initially activated by the EGFR pathway and that this activation requires Pnt function.

### Ap and Vg/Sd regulate the apDV CRM

**Fig 5:**
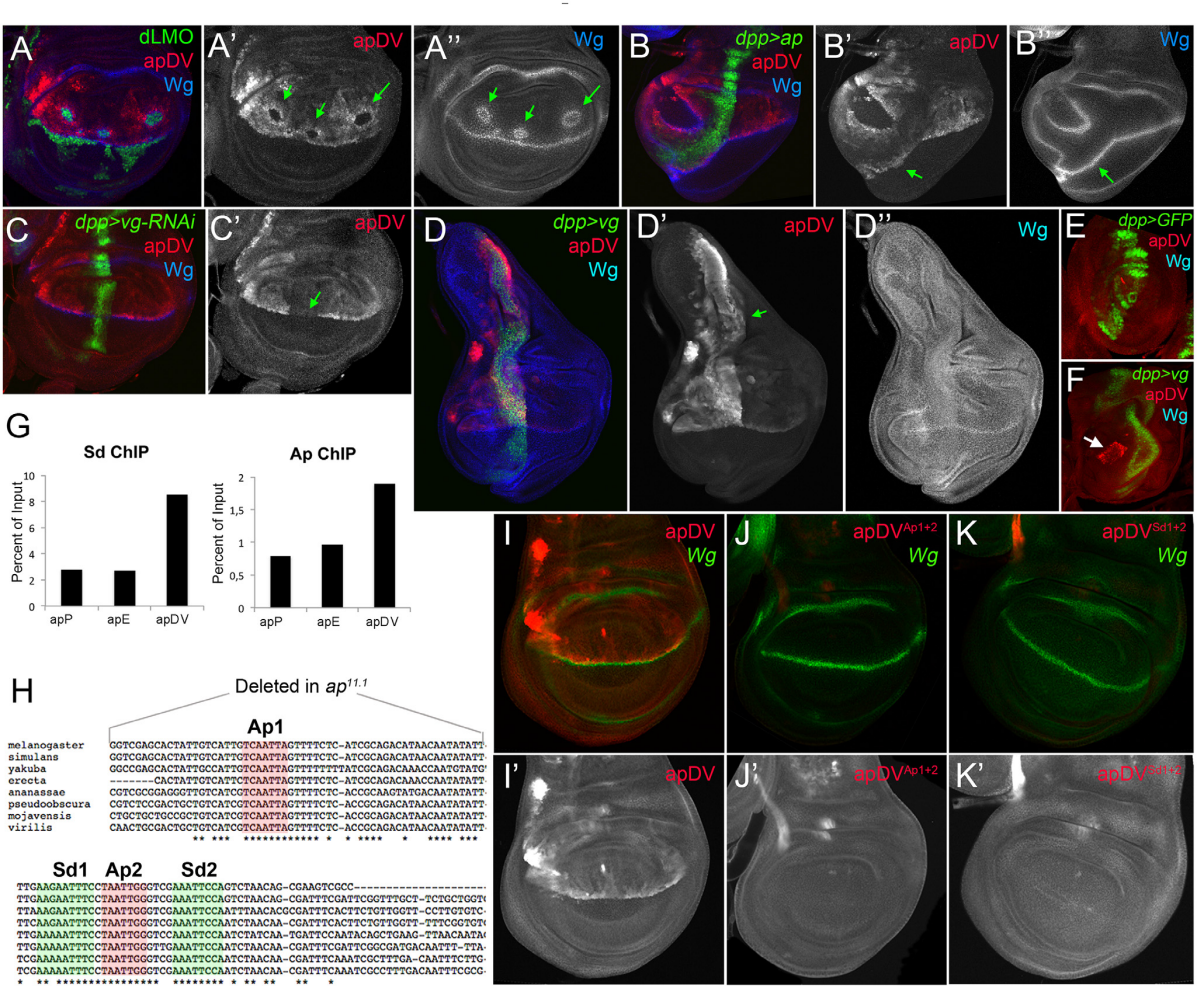
apDV is regulated by Ap and Sd/Vg. **(A)** In dLMO expressing clones (green), apDV-*lacZ* activity (red) is repressed. Wg (blue) is non-autonomously activated in cells surrounding the clones. Single channels are displayed for apDV-*lacZ* **(A’)** and Wg **(A”)**. Green arrows point to dLMO expressing clones. **(B)** *dpp*-*Gal4*; UAS-*GFP*, UAS-*ap* (green): upon ectopic Ap induction by *dpp-Gal4*, apDV-*lacZ* (red) and Wg (blue) are induced in ventral compartment cells. Single channels are displayed for apDV-*lacZ* **(B’)** and Wg **(B”)**. Green arrow points to ectopic apDV and *wg* expression. **(C)** *dpp*-*Gal4*; UAS-*GFP*, UAS-*vg*-RNAi: RNAi-induced knockdown of Vg in *dpp* domain (green). Wg is in blue. Note that apDV-*lacZ* expression (red) is strongly downregulated in the central part of the pouch. **(C’)** Single channel display of apDV-*lacZ*. Green arrow points to discontinuity in the apDV pattern. **(D)** *dpp*-*Gal4*; UAS-*GFP*, UAS-*vg*: ectopic *vg* expression induces apDV (red) along the *dpp* domain (green), but only in the dorsal compartment. Note that *wg* is not induced upon the ectopic expression of *vg*. Single channels are displayed for apDV-*lacZ* (**D’**; green arrow points to ectopic apDV-*lacZ*) and Wg **(D”)**. **(E-F)** *dpp*-*Gal4*; UAS-*GFP* **(E)** and *dpp*-*Gal4*; UAS-*GFP*, UAS-*vg* leg discs **(F)**: *dpp*-*Gal4*; UAS-*GFP* (green), apDV-*lacZ* (red) and Wg (blue) patterns are shown. Ectopic *vg* expression induces apDV activity in the distal domain of the leg disc (white arrow in **F**). **(G)** ChIP experiments with anti-Sd and anti-Ap antibodies. Quantifications of apP, apE and apDV DNA in immunoprecipitates demonstrate that Sd and Ap are preferentially bound to the apDV regulatory region. Representative enrichment values are shown for a single experiment that was conducted in triplicate. **(H)** DNA sequences of various *Drosophilae* species surrounding the identified Ap (red shade) and Sd (green shade) binding sites are shown. Note that Ap1 site is deleted in *ap* ^*11*.*1*^ flies. **(I-K)** Wing imaginal discs stained for Wg (green) and apDV (**I**, red), apDV^Ap1+2^ (**J**, red) and apDV^Sd1+2^ (**K**, red) activity. Mutation of the Ap sites **(J)** or Sd sites **(K)** results in loss of apDV activity. **(I’-K’)** Single channel pictures are depicted for each apE wild type and mutant condition. All constructs have been inserted in the same genomic location and images were obtained keeping the confocal settings constant.

While the apE element was activated in all *ap*-expressing cells in early second instar wing discs, the apDV element was induced only later and restricted to a subset of apE-positive cells (the wing pouch cells). Therefore, we tested whether the Ap protein itself is needed in an autoregulatory fashion for the restricted activity of the apDV element in the dorsal compartment. dLMO expressing clones cell-autonomously repressed the apDV element, while forced expression of *ap* in the ventral compartment cells ectopically activated it (Fig 5A and 5B). This suggests that Ap restricts the dorsal activity of apDV. Although *ap* is expressed in all dorsal wing disc cells, apDV is only active in dorsal wing cells close to the D/V boundary, which suggests additional input into this element. Therefore, we tested whether apDV activity is controlled by *wg* or *vestigial* (*vg*) [40], two key genes required for wing development. Downregulation or ectopic activation of the Wg pathway did not significantly affect apDV-*lacZ* expression (S4 Fig). However, knockdown of *vg* in the *dpp* domain eliminated apDV-*lacZ* expression (Fig 5C). Additionally, ectopic expression of *vg* strongly activated apDV in the dorsal compartment (Fig 5D). Remarkably, while apDV is not activated in the leg disc, forced expression of *vg* in this disc induced its activity in the distal domain of the leg, where a ring of endogenous *ap* expression has been described (Fig 5E and 5F)[16,41].

As a next step, we tested whether Ap and Scalloped proteins (Sd), the transcriptional companion of Vg, directly bind to the apDV CRM. Using Ap and Sd chromatin immunoprecipitation (ChIP), we found that Ap and Sd were significantly enriched at the apDV regulatory region in comparison to apE or apP (Fig 5G). Moreover, we identified two conserved consensus-binding sites for Sd as well as for Ap in the apDV region. Mutation of these sites completely eliminated apDV activity (Fig 5H–5K). Intriguingly, loss of one of these Ap binding sites likely contributes to the wing defects seen in the *ap* ^*11*.*1*^ mutant described previously (see Fig 1I).

Taken together, these results suggest that Ap and Vg/Sd directly regulate apDV in the wing pouch, with an Ap autoregulatory input restricting its activity to the dorsal compartment.

### Synergistic effect of apE and apDV with the *ap* promoter directs *ap* expression in the wing disc

**Fig 6:**
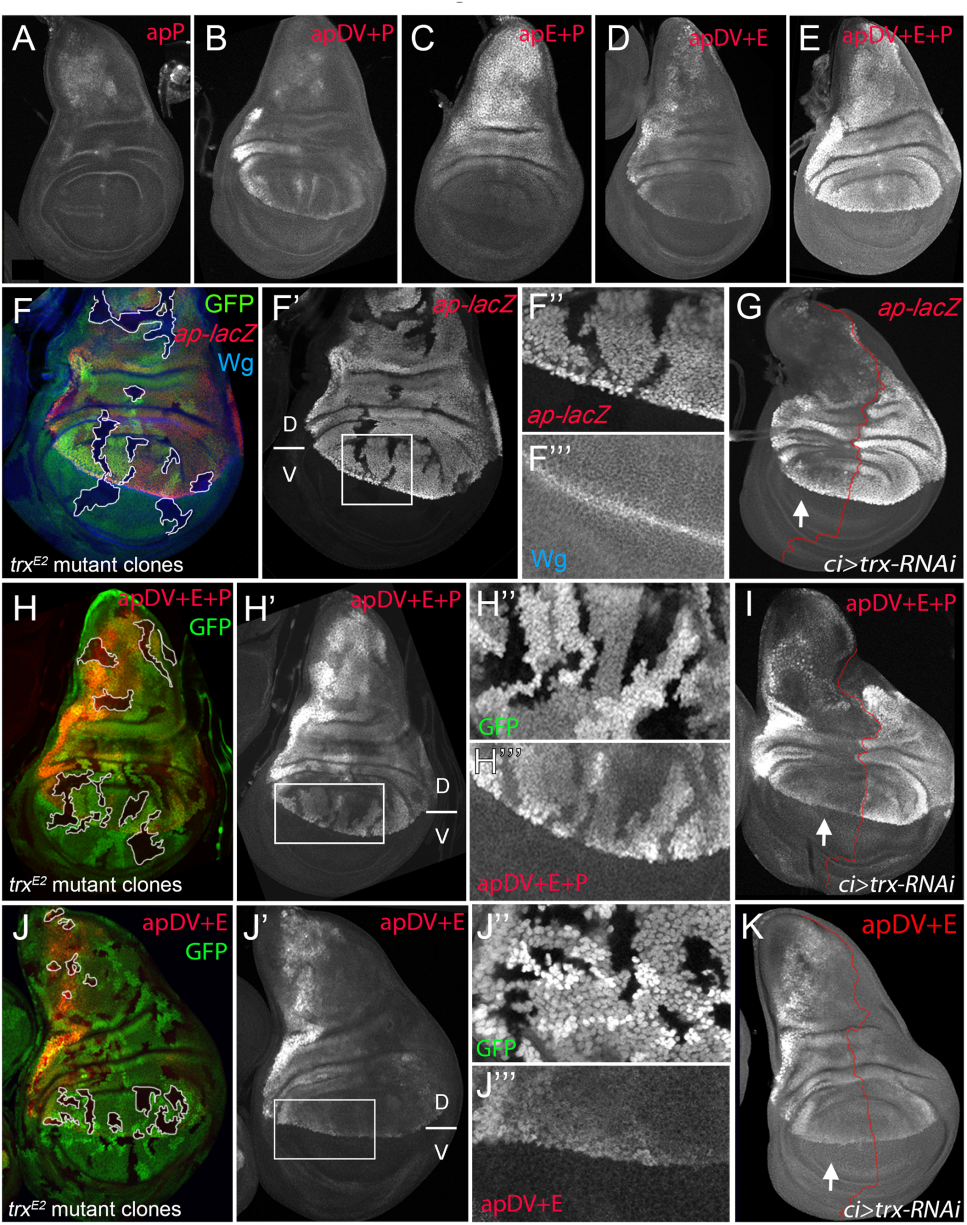
apP mediates *ap* expression maintenance and depends on Trx input (A-E) Third instar wing imaginal discs stained with α-βGal antibody to visualize *lacZ* activity. **(A)** apP activity is weak and not related to the endogenous *ap* expression pattern. **(B)** apDV+P activity is the sum of both elements. **(C)** The combination of apE+P leads to stronger and more homogeneous *lacZ* expression in the notum and hinge. Note that expression levels remain low in the dorsal wing pouch. **(D)** apDV+E activity is the sum of apDV and apE and does not reproduce the complete *ap* expression pattern. **(E)** Only the combination of apDV+E+P reproduces the endogenous *ap* pattern. All constructs were inserted in the same genomic location. **(F, H and J)** *trx* ^*E2*^ mutant clones were generated 48–72hrs AEL and are marked by the absence of GFP (in each disc, several of them are outlined in white). Discs were stained for Wg (blue) and *ap*-*lacZ* (red, **F**), apDV+E+P-*lacZ* (red, **H**) and apDV+E-*lacZ* (red, **J**). **(F’,H’,I’)** single channel image of *lacZ* staining. **(G, I, and K)** *ci*-*Gal4*; UAS-*trx*-RNAi: RNAi-induced knockdown of Trx-activity in the anterior wing disc compartment. Imaginal disc were stained for β-Gal protein. White arrow points to anterior wing compartment. **(G)** *ap-lacZ*: enhancer trap *ap* ^*rK568*^, **(I)** apDV+E+P-*lacZ* and **(K)** apDV+E-*lacZ*. **(F, F’)** *trx* ^*E2*^ mutant clones show downregulation of *ap* expression. **(F”** and **F”’)** Close-up of *ap-lacZ* and *wg* expression shown in **F’**. **(G)** Knockdown of Trx in the anterior compartment downregulates *ap*-*lacZ* expression. Note that the reduction of *ap*-*lacZ* is stronger in the notum and the wing pouch close to the hinge. **(H and H’)** *trx* ^*E2*^ mutant clones show downregulation of apDV+E+P-*lacZ* expression. (**H”** and **H”’**) Close-up of GFP and apDV+E+P activity in **H’**. **(I)** Knockdown of Trx in the anterior compartment (arrow) downregulates apDV+E+P-*lacZ* expression. As *ap-LacZ* in **(G)**, apDV+E+P activity is reduced in a spatial dependent manner. **(J and J’)** *trx* ^*E2*^ mutant clones show no effect on apDV+E-*lacZ* expression. (**J”** and **J”’**) Close-up of GFP and apDV+E activity in **J’**. **(K)** Reducing Trx in the anterior compartment has no effect on apDV+E-*lacZ* expression. D, dorsal and V, ventral.

We have identified two *ap* CRMs (apE and apDV) that, when combined in a reporter assay, partially recapitulated *ap* expression in the wing disc (see Fig 3F), suggesting that other CRMs are needed for full expression. Since PRE-containing sequences are necessary for correct *ap* expression and proper wing development (Fig 1C), we tested if a region around the *ap* TSS including the PRE, named apPRE (apP), had an impact on the activity of the distal *ap* CRMs (Fig 3A). On its own, the apP drove weak expression in the wing disc in a pattern not related to the characteristic *ap* expression (Fig 6A). When placed together in a reporter construct with either apDV or apE (resulting in apDV+P-*lacZ* or apE+P-*lacZ*), the activity of the resulting reporter gene construct was the sum of both elements and did not reproduce faithfully the *ap* expression pattern (Fig 6B and 6C). Interestingly, when the three CRMs were placed together, the expression of the apDV+E+P-*lacZ* in third instar wing discs was more accurate than the expression of the previous CRMs combinations or the apDV+E-*lacZ* and more precisely reproduced the expression pattern of *ap* (compare Fig 6D with 6E).

Therefore, we tested whether these *ap* CRM combinations placed next to *ap* cDNA were sufficient to rescue wing development in an *ap* mutant background. As expected, apE+P*-apcDNA* was partially able to rescue wing growth, but completely lacks the D/V margin, whereas an apDV+P-*apcDNA* transgene, that lacks the apE enhancer, did not rescue wing formation (S5 Fig). Interestingly, the apDV+E+P-*apcDNA* transgene rescued wing formation in *ap* mutants. Although the rescue was not fully wild type, a clear wing margin was observed in wing discs and adult wings (S5 Fig).

In summary, we have identified three *ap* CRMs that, only when combined, can accurately reproduce the endogenous *ap* expression pattern in the wing imaginal disc.

### Trx maintains robust *ap* expression via the apP element

It has been proposed that PcG proteins repress *ap* expression in ventral wing disc cells and that sequences around the *ap* TSS could function as a PRE [30]. However, the role of TrxG proteins in the control of *ap* expression in wing imaginal discs has not been tested previously. Therefore, we generated *trx* ^*E2*^ mutant clones and studied *ap* expression with a *lacZ-*enhancer trap inserted immediately 5’ to the *ap* TSS (*ap* ^*rK568*^). We found that cells devoid of *trx* function show reduced *ap-lacZ* expression (Fig 6F). To analyze this result in more detail, we reduced *trx* mRNA levels in the anterior wing disc compartment (*ci-Gal4>trx-RNAi*) and compared the levels of *ap-lacZ* expression with the posterior control compartment (Fig 6G). Consistent with *trx* mutant clones, *ap-lacZ* expression was strongly reduced in the anterior compartment, although the reduction was more prominent in the notum and in the dorsal wing pouch close to the hinge.

To genetically confirm that the *ap* PRE (apP) functions as a Trithorax response element (TRE), we eliminated or downregulated Trx activity and analyzed the expression of the apDV+E+P*-lacZ* reporter construct. In *trx* ^*E2*^ mutant clones, apDV+E+P*-lacZ* levels were strongly reduced, as it was the case for *ap-lacZ* (Fig 6H). In contrast, the same construct without the *ap* promoter, apDV+E*-lacZ*, was not altered in these *trx* mutant clones (Fig 6J). Accordingly, reducing the levels of Trx in the anterior compartment cells (*ci>trx-RNAi*) did not affect expression of apDV+E*-lacZ* (compare Fig 6K with 6I). Interestingly, the expression pattern of apDV+E+P*-lacZ* was strongly reduced upon Trx downregulation and resembled the pattern of wild type apDV+E*-lacZ* (the same construct without the apP, compare Fig 6G and 6I with 6K).

Altogether, our results suggest that the *ap* promoter region behaves as a PRE/TRE providing the information required to maintain *ap* expression.

### Direct and continuous contact of the apDV and apE CRMs together with the apP element for *ap* maintenance

Classical transvection experiments usually deal with chromosomes harboring genes lacking either a functional promoter region or a functional enhancer. For combinations of members of the two groups, intragenic complementation can be observed, i.e. the corresponding phenotype is much less severe than seen in allelic combinations involving only one or the other group [42,43]; reviewed in [44]. We have previously reported that transvection is at work at *ap* [35]. For example, *ap* ^*DG12*^ */ap* ^*DG1*^ flies have no wings because both alleles delete wing enhancer apE. The same phenotype is observed in *ap* ^*t11b*^ */ap* ^*DG8*^ flies because both alleles remove the promoter region as well as the 5’ end of *ap*. In contrast, the wing phenotype of *ap* ^*t11b*^ */ap* ^*DG1*^ flies is much improved (S7B Fig). Models for transvection posit that the apE and apDV enhancers on chromosome *ap* ^*t11b*^ can activate the transcription machinery of the functional *ap* gene on chromosome *ap* ^*DG1*^. However, the *ap* ^*t11b*^ */ap* ^*DG1*^ wings are consistently less well formed than those obtained from *ap* ^*DG3*^ */+* flies (see S7 Fig). These observations suggest that the apP region on the one hand and the two enhancers on the other interact more efficiently if they are located *in cis*.

**Fig 7:**
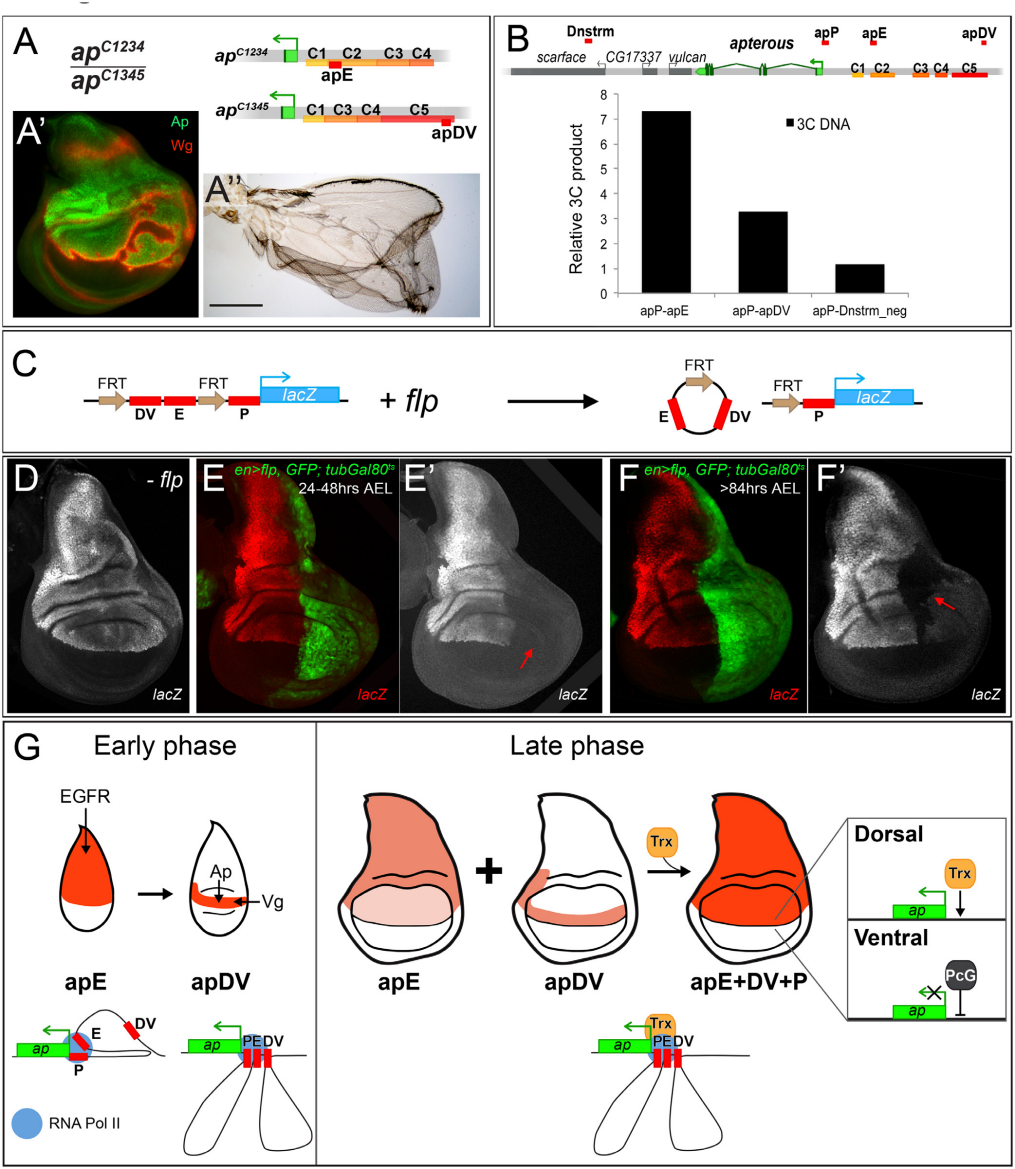
Evidence for genetic and physical interaction between apDV, apE and apP. **(A)** At the top of the panel, the genetic constitution of *ap* ^*C1234*^ */ap* ^*C1345*^ flies is shown. Note that apE and apDV are present in *trans* and that apP (not indicated) is present on both chromosomes. **(A’)** Ap (green) in the wing disc is uneven leading to derepression of *wg* in cells with no Ap (red). **(A”)** Wings of *ap* ^*C1234*^ */ap* ^*C1345*^ flies frequently show wing patterning defects and outgrowths. **(B)** At the top of the panel, a schematic representation of the *ap* genomic locus and several flanking genes is shown. C1–C5 indicates the conserved homology regions. Red bars above the chromosome represent the different regions tested for direct interaction with apP using the 3C technique. A region downstream of *ap*, named Dnstrm, was used as a negative control. The diagram in the lower part of the panel summarizes the 3C data. In whole third instar larvae, apE and apDV elements are more frequently close to apP than a control DNA element located downstream (Dnstrm) the *ap* genomic locus. **(C)** Diagram of the FRT*-*apDV+E*-*FRT*+*P*-lacZ* reporter gene is depicted. Upon *flp* induction, the apDV+E cassette is deleted. The *lacZ* reporter remains under the control of apP only. **(D-F)** Expression of the FRT*-*apDV+E*-*FRT*+*P*-lacZ* reporter gene in third instar wing imaginal discs in the absence of *flp* **(D)** or after *flp*-induction at different times of larval development **(E-F)**. Controlled *flp*-induction in the posterior pouch compartment (red arrow) was achieved and monitored in an *en*-*Gal4*; UAS-*flp*; UAS-*GFP*, *tubGal80* ^*ts*^ background. Wing discs were stained for *lacZ* (red) and GFP (green). **(D)** *lacZ* pattern without *flp*-induction resembles wild type *ap* expression. **(E)** *flp*-induction 24–48hrs AEL: deletion of the apDV+E cassette results in loss of *lacZ* expression in the posterior compartment. **(F)** *flp*-induction >84hrs AEL: Loss of *lacZ* expression in the posterior compartment after late *flp-*induction demonstrates the continuing requirement of apE and apDV. **(G)** *ap cis*-regulatory model for the establishment of Dorso-Ventral identity in the wing imaginal disc. During the early phase of *ap* activation, the EGFR pathway triggers *ap* expression via the apE CRM that directly interacts with apP. A few hours later, apDV is activated in dorsal cells close to the D/V boundary. It is activated by Vg/Sd in the future wing cells but its activity is restricted dorsally by Ap itself. apDV is also recruited to apP. In the late phase, apP maintains *ap* expression through Trx input in dorsal cells. Persistent *ap* transcription is required for the generation of a dorsal lineage compartment. It is dependent on the permanent presence of the apE and apDV enhancers and their continuing interaction with apP. PcG proteins repress *ap* in ventral compartment cells.

In our study, we have shown that the two *ap* wing enhancers are clearly separable units: (1) they lie ~10 kb apart and (2) the activity of apE is essential for auto-regulatory activation of apDV. From these premises, one would not *a priori* expect that the two enhancers must be *in cis* for full function. However, several allelic combinations containing only one or the other enhancer element (apE or apDV) generated discs and adult wings with defects at the D/V boundary: similar results were obtained for genotypes *ap* ^*C1234*^ */ap* ^*C1345*^, *ap* ^*DG14*^/*ap* ^*DG12*^, *ap* ^*C2*^/*ap* ^*DG12*^ or when a *su(Hw)* insulator element was inserted between apDV and apE in *ap* ^*f00451*^/*ap* ^*DG3*^ animals (Fig 7A and S7 Fig). Our transvection studies suggest that all three CRMs need to be in *cis* to fully rescue wing development.

To better understand how the synergy between the three regulatory elements is achieved, we used chromosome conformation capture (3C) [45], which allowed us to test *in vivo* whether there is direct physical contact between the apE and apDV CRMs with the apP. Indeed, as seen in Fig 7B, we found that in whole third instar larvae, apP preferentially contacted the apE and apDV elements, and did so more frequently when compared to sequences outside the *ap* genomic locus. This suggests that the distal apE and apDV regions are in close physical proximity to apP *in vivo*.

Next, we tested whether apDV and apE CRMs are required either continuously or only transiently to direct *ap* expression during wing disc development. To distinguish between these two possibilities, we generated an apDV+E+P*-lacZ* construct, in which the apDV+E is flanked by FRT sequences (FRT*-*apDV+E*-*FRT*+*P*-lacZ*, Fig 7C). This allowed us to remove the apDV+E cassette at different time points of wing development using Flp-mediated recombination [46,47] (see Materials and Methods). Deletion of apDV+E early in development in the posterior compartment completely abolished reporter expression compared to anterior control cells (compare Fig 7D and 7E). Deletion of the apDV+E at later stages also strongly decreased reporter gene expression (Fig 7F).

In summary, these experiments suggest that there is a direct contact between the apE and apDV with the *ap* promoter and that these three elements need to be *in cis* throughout wing disc development to confer optimal *ap* expression.

## Discussion

The selector gene *ap* encodes for a transcription factor that confers dorsal identity in the wing imaginal disc. A precise border of *ap*-expressing and non-expressing cells is absolutely necessary for wing growth and pattern formation. Although the role of *ap* as a dorsal selector gene has been extensively studied, how its specific spatial expression pattern is brought about during wing development has remained unclear. In this work, we have used complementary strategies to identify and molecularly characterize the endogenous CRMs that regulate *ap* expression during wing development.

### *ap cis*-regulatory logic for Dorso-Ventral identity in the wing imaginal disc

Our genetic and *cis*-regulatory analysis provides information about the logic of *ap* expression during wing development. We propose that *ap* expression is controlled by at least three CRMs that act in combination (Fig 7G). The first element, apE is the earliest to be activated in proximal wing disc cells via the EGFR pathway; its expression subsequently weakens in the wing pouch. Deletion of this early enhancer (e.g. *ap* ^*DG12*^ or *ap* ^*C1345*^) completely abolishes wing formation. The asymmetry of *ap* expression to the proximal domain of the wing disc is probably due to the localized activation of the EGFR pathway by its ligand Vn and a distal repression by Wg signaling [26,48–50]. We have genetically and molecularly confirmed the initial activation of the apE by the EGFR pathway; however, other inputs are required for the continuous activation of this CRM in later wing discs.

A few hours after apE activation, a second CRM, apDV, is activated in a subset of apE positive cells. In contrast to apE, apDV is restricted to the dorsal-distal domain of the wing pouch by direct positive inputs from Ap and Vg/Sd (Fig 7G). The direct Ap autoregulatory input defines the time window when the apDV element is activated; apDV can only be active after the induction of Ap by the early enhancer (apE). It has been shown that Ap induces *vg* expression by triggering Notch signaling at the D/V boundary [20,21,48,51,52]. Thus, the (direct) input of Vg/Sd on apDV can be regarded as an indirect positive autoregulation, which delimits the spatial domain where apDV can be actived. Consequently, the interface of Ap and Vg expression defines the region of apDV activity via positive autoregulation.

The third *ap* CRM is the *ap* PRE/TRE region (apP), that, when deleted, leads to a strong hypomorphic wing phenotype (*ap* ^*c1*.*2b*^). The apP requires Trx input and maintains *ap* expression when placed in *cis* with the apDV and apE CRMs (Fig 7G). Only the combination of the three CRMs faithfully reproduces *ap* expression in the wing disc. Moreover, our regulatory *in locus* deletion and *in situ* rescue analysis provide strong functional relevance for these CRMs.

Ultimately, this cascade of *ap* CRMs provides a mechanism to initiate, refine and maintain *ap* expression during wing imaginal disc development, in which the later CRMs depend on the activity of the early ones (Fig 7G). A similar mechanism has been described for *Distal-less* (*Dll*) regulation in the leg primordia where separate CRMs trigger and maintain *Dll* expression in part by an autoregulatory mechanism [46,53].

It has been proposed that positive autoregulation may help to maintain the epigenetic memory of differentiation [54]. In the case of *ap*, we demonstrate that autoregulation works in conjunction with a PRE/TRE system; this might make the system very robust and refractory to perturbations.

### The role of *ap* promoter in maintenance

ChIP experiments have shown that many developmentally important genes are associated with a promoter proximal PRE as found at *ap* [30]. The role of such a PRE has been studied at the *engrailed* (*en*) locus. It has been demonstrated that in imaginal discs, the promoter as well as the promoter proximal PRE are important for the long-range action of *en* enhancers [55,56]. These authors propose that this PRE brings chromatin together, allowing both positive and negative regulatory interactions between distantly located DNA fragments.

Our results indicate that sequences around the transcription start of *ap* (apP) may serve a similar function. First, this element, when placed in *cis* with the *ap* CRMs (apE and apDV), maintains the *ap* expression pattern and keeps reporter gene expression off in cells where low or no activity of apDV and apE has been observed. Second, in the absence of *trx*, the expression of *ap* and apDV*+*E*+*P*-lacZ* is strongly reduced. All these data suggest that sequences within the apP integrate Trx input, thereby maintaining *ap* expression in a highly proliferative tissue such as the wing disc. Interestingly, *trx* mutant clones were not round and did not show ectopic *wg* activation (Fig 6F), which is a hallmark of *ap* loss-of-function clones. This suggests that in *trx* mutant clones enough Ap protein is still present to maintain *wg* expression off. However, we found derepression of the ventral-specific integrin *αPS2* in *trx* mutant clones in the wing pouch as previously described for *ap* mutant clones [14] (S6 Fig).

It has been suggested that TrxG proteins could act passively antagonizing PcG silencing, rather than playing an active role as co-activators of gene transcription [57,58]. For example, *Ubx* expression in the leg and haltere does not require Trx in the absence of Polycomb repression [59]. We tested these possibilities and generated *trx* mutant clones that were also mutant for the PcG member *Sex combs on midlegs* (*Scm*). Dorsally-located *Scm* ^*-*^ *trx* ^*-*^ double mutant clones still downregulate *ap-lacZ* expression while ventral-induced ones are unable to derepress *ap-lacZ* as we observed for *Scm* ^*-*^ single mutant clones (S6 Fig). Therefore, our results, in addition with previous findings by Oktaba *et al* [30], suggest that TrxG maintains *ap* expression in dorsal cells, while *ap* expression is repressed in the ventral compartment by PcG proteins. Moreover, it has been shown that the sequences around the *ap* transcription start, including the PRE, are occupied by PcG complexes PRC1 and PRC2, as well as Trx [30,60].

### Direct and continuous interactions between the apE and apDV with the apP

Enhancers-promoter interactions initiate transcription but their dynamics during development have remained poorly understood. Our chromosome conformation capture (3C) experiment provides evidence for the direct interaction between the *ap* CRMs apE and apDV with the maintenance element encoded by the apP. Beyond this, we also find that these elements cooperate continuously during wing development. Our flip-out experiments, in which we removed the apDV and apE CRMs at different time points, suggest that these elements need to be present continuously to ensure correct *ap* expression. Additionally, flies carrying apE only on one chromosome and apDV only on the homologue were unable to fully rescue wing development suggesting that these CRMs need to be in *cis*. It is conceivable that *in cis* configuration of the three *ap* CRMs facilitates and stabilizes enhancer-promoter looping. It could also help to rapidly establish relevant chromatin contacts after each cell division. These results are in accordance with previous observations, in which constant interactions between *ap* enhancers and promoter during embryogenesis have been described [61]. Our results extend these observations to the wing disc, a highly proliferative tissue, where the expression of the trans-factors that regulate the activity of the apE and apDV is very dynamic. This raises the question on how this contact is re-assembled over many cell generations. It is possible that some epigenetic modifications are laid down in the activated apE and apDV CRMs, which are then inherited during cell divisions to ensure contact with apP. Studies of the chromatin status of these elements will be required to fully understand this process.

### Developmental transcriptional regulation during tissue growth

A key question in developmental biology is how transcriptional regulation is coupled to tissue growth to precisely regulate gene expression in a spatio-temporal manner. For example, during *Drosophila* leg development, initial activation of the ventral appendage gene *Dll* by high levels of Wg and Dpp initiates a cascade of cross-regulation between Dll and Dachshund (Dac) and positive feedback loops that patterns the proximo-distal axis [46,62]. Other mechanisms to expand gene expression patterns depend on memory modules such as PREs, as it is the case for the Hox genes or other developmental genes like *hh* [63–65]. To direct wing formation, expression of *ap* in the highly proliferative tissue of the wing disc must be precisely induced to generate and maintain the D/V border. Our in-depth analyses at the *ap* locus provide a functional and molecular explanation of how expression of this dorsal selector gene is initiated, refined at the D/V border, and maintained during wing disc development. We propose that this three-step mechanism may be common for developmental patterning genes to make the developmental program robust to perturbations.

## Materials and Methods

### Stocks used in this study

Flies were grown on standard cornmeal agar. *ap-lacZ* (*P{PZ}ap* ^*rK568*^), *ap*-*Gal4*, *ap* ^*UGO35*^, *trx* ^*E2*^, *Scm* ^*D1*^, *trx* ^*E2*^ *Scm* ^*D1*^ (gift from Jürg Müller)[59], *EGFR* ^*tsa*^, UAS-*EGFR* ^*λtop4*.*2*^, UAS-*Raf* ^*Dn*^ UAS-*arm* ^*S10*^, UAS-*TCF* ^*DN*^, UAS-*vg*, UAS-*dLMO*, UAS-*ap*, *dpp*-Gal4; UAS-*GFP*, *ci*-Gal4; UAS-*GFP*, *tubGal80* ^*ts*^. *act5C*>*stop*>*lacZ*; UAS-*flp*, *P{hsFLP}12*, *y* ^*1*^ w*, *TM3*, *ry* ^*RK*^ *Sb* ^*1*^ *Ser* ^*1*^ *P{Δ2–3}99B*, *P{EPgy2}l(2)09851* ^*EY06365*^, *al* ^*1*^ *b* ^*1*^ *c* ^*1*^ *sp* ^*1*^, *y* ^*1*^ *w* ^*67c23*^; *noc* ^*Sco*^ / CyO, *P{Crew}DH1*, y^1^ w*; *Mi{y[+mDint2] = MIC}MI00964*, y^1^ w*; *Mi{y[+mDint2] = MIC}MI02330/SM6a* as well as all the Janelia Farm Gal4 drivers were obtained from the Bloomington Drosophila Stock Center except as indicated. These are described in the Fly light data base (http://flweb.janelia.org/cgi-bin/flew.cgi): 1-GMR_39E04, 2-GMR_42A06, 3-GMR_42D11, 4-GMR_41B09, 5- GMR_41E03, 7-GMR_42B11, 8-GMR_41D11, 9-GMR_41D03, 10-GMR_40H04, 11-GMR_39B07, 12-GMR_40A08, 13-GMR_39G10, 14-GMR_ 39C09, 15-GMR_40A07, 16-GMR_41A02, 17-GMR_41C10. For the lineage analyses of the Janelia lines we used the *act5C>stop>lacZ*; UAS-*flp* [47]. UAS-*vg*-RNAi, UAS-*sd*-RNAi and UAS-*trx*-RNAi are available at the Vienna Drosophila Resource Center (VDRC). RNAi knock-down experiments were performed in a UAS-*Dcr–2* background. *en-Gal4; UAS*-*flp*, *UAS*-*GFP* was a gift from Laura Johnston. *PBac{RB}e01573*, *ap* ^*f08090*^ (*PBac{WH}f08090*),*ap* ^*f00878*^ (*PBac{WH}f08090*) and *ap* ^*f00451*^ (*PBac{WH}f00451*) were purchased from the Exelixis stock collection at Harvard Medical School. *y w M{vas-int*.*Dm}zh-2A*, a stock producing *ФC31*-integrase under the control of the vasa promoter, and docking site *M{3xP3-RFP*.*attP}zh-86Fb* were obtained from Johannes Bischof [66]. The *GFP* knock-in allele *ap* ^*GFP*^ is described in Caussinus *et al*, 2012 [67]. *ap* ^*MM*^ and *ap* ^*MM-Mcp*^ have been described previously [35]. They contain a P-element insertion ~400 bp upstream of the *ap* TSS. The ΦC31-integrase platforms *ap* ^*attPΔCDS*^ and *ap* ^*attPΔEnh*^ used for the *in situ* rescue system are described in detail in Caussinus *et al*, 2012 and Bieli *et al*, 2015, respectively [25,67]. The generation of all deficiencies shown in Fig 1A and 1B is described below.

Adult wings were dissected and mounted in Hoyer’s and baked at 58°Celsius for a few hours. Pictures were taken with a Nikon Microphot-FXA microscope with a Sony NEX-5RK digital camera.

The notums of adult flies were photographed with a Leica M125 binocular equipped with a Leica DFC420C camera.

### Generation of deletions

*Df(2R)ap* ^*DG1*^ is described in Gohl *et al*, 2008, where it is called *ap* ^*DG*^ [35].

*Df(2R)ap* ^*DG3*^, *Df(2R)ap* ^*DG8*^ and *Df(2R)ap* ^*DG11*^ are described in Bieli *et al*, 2015 [25].

*Df(2R)ap* ^*12*.*1*^, *al b* was obtained in an attempt to isolate male-recombination events to the right of P-element insertion *ap* ^*MM-Mcp*^. Molecular characterization identified the proximal breakpoint in a P-element insertion hot spot at the 5’ end of the *vulcan* gene (Genome release R6 FB2015_01: 2R:5702133). It also verified the integrity of ap^MM-Mcp^ at its original insertion site. This deletion is referred to as *Df(2R)ap* ^*12*.*1-Mcp*^, *al b*. In order to delete the *Mcp* element located between 2 loxP sites on *ap* ^*MM-Mcp*^, *Df(2R)ap* ^*12*.*1-Mcp*^, *al b* was treated with *Cre* recombinase [68] and *Df(2R)ap* ^*12*.*1*^, *al b* was obtained. Homozygous flies of this genotype make it to the pharate adult stage. Dissected individuals have neither wings nor halteres. In this study, *Df(2R)ap* ^*12*.*1*^, *al b* is referred to as *ap* ^*12*.*1*^. Note that it is associated with a FRT site left within *ap* ^*MM*^.

The following 6 deletions were created by *flp*-mediated recombination [69] between 2 FRT sites located *in trans* to each other in 2 different transposons (below, their names are indicated in parenthesis; their positions within the *ap* locus is depicted in Fig 1A):

*Df(2R)ap* ^*DG16*^, *al b* (*ap* ^*12*.*1*^; e01573). Referred to in the text as *ap* ^*DG16*^. This chromosome is deficient for *vulcan* and *ap*. Homozygous *ap* ^*DG16*^ flies are pharate adult lethal. Dissected individuals have neither wings nor halteres.

*Df(2R)ap* ^*DG2*^ (f08090; ap^MM-Mcp^). Referred to in the text as *ap* ^*DG2*^. Note that *Mcp* is lost upon flp-mediated recombination and that this deletion is associated with an array of Su(Hw) binding sites originating from f08090.

*Df(2R)ap* ^*DG15*^ (*ap* ^*EE23*.*9*^; e01573). Referred to in the text as *ap* ^*DG15*^. *ap* ^*EE23*.*9*^ (as well as *ap* ^*EE29*.*19*^) is a ΦC31-integrase mediated insertion of a plasmid containing *mini-white*, *FRT* and *mini-yellow* in docking site *MI00964* [70].

*Df(2R)ap* ^*DG6*^, *al* (ap^D5f.1^; f00451). Referred to in the text as *ap* ^*DG6*^. *ap* ^*D5f*.*1*^ is a ΦC31-integrase mediated insertion of a plasmid containing *mini-white*, *FRT* and *mini-yellow* in docking site *ap* ^*c1*.*4b*^ [25]. Note that this deletion is associated with an array of Su(Hw) binding sites originating from f00451. *ap* ^*DG6*^ flies have neither wings nor halteres. These phenotypes are not modified in a *su(Hw)* ^*-*^ background.

*Df(2R)ap* ^*DG12*^ (*ap* ^*EE29*.*19*^; *ap* ^*DD8*.*1*^). Referred to in the text as *ap* ^*DG12*^. *ap* ^*DD8*.*1*^ (as well as *ap* ^*DD35*.*34*^) is a ΦC31-integrase mediated insertion of a plasmid containing *mini-white*, *FRT* and *mini-yellow* in docking site *MI02330* [70].

*Df(2R)ap* ^*DG14*^ (*ap* ^*DD35*.*34*^; e01573). Referred to in the text as *ap* ^*DG14*^.

*Df(2R)ap* ^*c2*.*73c*^: this short deletion was obtained by direct gene conversion [71,72]. A detailed account on our experimental approach is given in Bieli *et al*, 2015 [25]. *ap* ^*c2*.*73c*^ was obtained according to the exact same procedure as *ap* ^*c1*.*4b*^, except that the left homology arm on the gene conversion template plasmid was only 502 bp long, leading to a 397 bp deletion just proximal to *ap* ^*MM*^. Our gene conversion approach also introduced a cassette consisting of a GFP reporter driven by a minimal *hsp70* promoter flanked by two inverted attP sites for Recombination Mediated Cassette Exchange (RMCE) [73].

The following five deletions were obtained by imprecise excision of insert *ap* ^*MM*^ during the generation of gene conversion events *ap* ^*c1*.*4b*^ and *ap* ^*c2*.*73c*^. In all five cases, the deletion extends only to the left of *ap* ^*MM*^.

*Df(2R)ap* ^*c1*.*78a*^: 12 bp are left between the break points, 8 of them can be identified as belonging to the end of the P-element 3’ foot. Referred to in the text as *ap* ^*c1*.*78a*^.

*Df(2R)ap* ^*c2*.*58c*^: the most 3’ ~1.6 kb of *ap* ^*MM*^ are left at the break point, including the wing enhancer of the *yellow* gene. Referred to in the text as *ap* ^*c2*.*58c*^.

*Df(2R)ap* ^*t11b*^: the terminal 17 bp of the P-element 3’-foot are left between the breakpoints. Referred to in the text as *ap* ^*t11b*^.

*Df(2R)ap* ^*c1*.*2b*^: the intact *ap* ^*MM*^ insert is left at the break point. Referred to in the text as *ap* ^*c1*.*2b*^.

*Df(2R)ap* ^*c1*.*60c*^, *sp*: the intact *ap* ^*MM*^ insert is left at the break point. This small deletion can be maintained as a homozygous stock and most wings look wild-type. Referred to in the text as *ap* ^*c1*.*60c*^.

Finally, *Df(2R)ap* ^*11*.*1*^, *c sp* was obtained by transposase treatment of *EY06365*, a P-element inserted in the 5’ end of *l(2)09851*, the gene immediately distal to *ap* (see Fig 1A). In an attempt to isolate deletions extending proximal to *EY06365*, dysgenic males of the genotype *y w*; *al ap* ^*DG3*^ *{w* ^*+*^ *} + + +* / *+ + EY06365{y* ^*+*^ *w* ^*+*^ *} c sp*; TM3, Sb, Δ2–3 / + were crossed with *y w*; *al b c sp* / SM6, *al sp* females. Progeny was screened for candidates with no eye colour, *y* ^*+*^ body colour and carrying the c and sp markers. 2 of the candidate chromosomes (isolation numbers 11.1 and 34.1) gave rise to notched wings *in trans* to *ap* ^*DG3*^, a phenotype reminiscent of weak *ap* alleles. SM6 balanced stocks were established. Homozygous flies readily hatch and show no or only very weak wing phenotypes. Molecular characterization of *EY06365* and the 2 candidates detected in all three a ~400 bp LTR of the *springer* retrotransposon at position 2R:5751931 (Genome release R6 FB2015_01). *EY06365/ap* ^*DG3*^ flies have normal wings indicating that the LTR doesn’t have phenotypic consequences. Furthermore, remarkably similar rearrangements could be detected in candidates 11.1 and 34.1: *EY06365* has relocated into exactly the same site in the hybrid piggyBac present on *ap* ^*DG3*^ (obtained by *flp*-mediated recombination between *FRT*s in *f08090* and *e01573*) in between *mini-white* and *FRT*. On the proximal side of the relocated *EY* element and next to the 3’ P-element foot, 11.1 contains ~100 bp of DNA originating from the 5’ end of *CR44953*, while 34.1 contains ~200 bp of DNA originating from the *rosy* locus. These insertions of heterologous DNA normally found on chromosome arm 3R are abutted by a ~1.7 kb deletion that extends to the left into the *apterous* region, 11.1 removing 8 bp more than 34.1. The two rearrangements are referred to as *ap* ^*11*.*1*^ and *ap* ^*34*.*1*^. Apart from these, two other very similar rearrangements associated with smaller deficiencies were isolated. Their names are *ap* ^*72*.*2*^ and *ap* ^*62*.*3*^. Their distal break point is the same as for *ap* ^*11*.*1*^ and *ap* ^*34*.*1*^ but they are smaller: 657 bp and 480 bp are missing, respectively. In both cases, hemizygous flies have normal wings, implying that the different position of their proximal deletion break is responsible for the wing phenotype observed for *ap* ^*11*.*1*^ and *ap* ^*34*.*1*^. These observations map the distal end of the *ap* regulatory domain to a 1 kb interval between the proximal ends of deficiencies *ap* ^*11*.*1*^ and *ap* ^*72*.*2*^.

### Generation of α-Ap antibody

DNA corresponding to amino acids 312 to 469 of *ap* cDNA clone HL02012 (DGRC, Indiana University) was amplified by PCR and cloned into pET22b(+) bacterial expression vector (Novagen) via NcoI and NotI sites. This fragment contains the Ap homeodomain (apHD), which is shared by all different Ap isoforms. The pelB leader sequence of pET22b vector was subsequently removed via mutagenesis PCR [74], resulting in the final expression plasmid pETapHD. BL21(DE3) bacteria (NEB) were transformed with pETapHD, grown to OD_600nm_ 0.6. T7 polymerase was induced with 0.1 mM IPTG. The protein was produced overnight at 18°C. Bacterial cells were lysed using a French press, then the lysate was loaded on a HisTrap HP column (GE Healthcare Life Sciences). apHD was purified with an ÄKTA HPLC machine. 3 mg of pure apHD were sent to Perbio Sciences Switzerland, where two rabbits were immunized. After 80 days, the serum of one positive rabbit was used to perform affinity purification of polyclonal antibody pool (final concentration: 0.67 mg/ml). For imaginal disc staining, the antibody is used at a dilution of 1:1000–2000.

### Cloning of *in situ* rescue constructs

First, fragments C1 (size: 1.6 kb), C2 (3.6 kb), C3 (2.5 kb), C4 (1.6 kb), C5 (5.3 kb), C5A (3.8 kb), C5B (600 bp) and OR463 (463 bp) were amplified by PCR from clone BACR45O18 (Berkeley Drosophila Genome Project). The PCR primers had AvrII or XmaI sites overhangs, respectively (see S1 Table for Primer sequences). PCR-fragments were cut with AvrII and XmaI and subcloned into pBS KSII(+) vector, in which the XbaI site had previously been mutated into an AvrII site. Primers containing the XmaI site additionally had a SpeI site. AvrII and SpeI produce compatible sticky ends, which –when ligated- cannot be cut again by any of these enzymes. To combine the different fragments in the desired order, the following strategy was used: one fragment was cut out with AvrII and XmaI, and cloned into another pBSKSII subclone, that had a different fragment, via SpeI and XmaI sites. In the new subclone two different fragments were combined, which could be cut out again via AvrII and XmaI sites and cloned into another SpeI/XmaI cut plasmid. Subsequently, the combined fragments were cut out with AvrII/XmaI and cloned into AvrII/AgeI cut pEnh-Reentry plasmids, resulting in the final pEnh-Reentry constructs. Detailed description of the pEnh-Reentry plasmid can be found elsewhere [25]. Transgenic flies were obtained by injecting these plasmids (300ng/μl final concentration) into *y w M{vas-int*.*Dm}zh-2A; ap* ^*attPΔEnh*^/*CyO* embryos and stocks were established according to standard genetic practice [75].

### Cloning of *ap* coding sequence *in situ* rescue constructs

*ap* cDNA was amplified from clone HL02012, the *ap* promoter region was PCRed from BAC clone BACR45O18 (Berkeley Drosophila Genome Project). The two fragments where combined by fusion PCR, and subcloned into pCR-XL-TOPO (Invitrogen). The *ap* promoter-cDNA fusion fragment was cloned into pCDS-Reentry vector [67] via NotI and AscI sites, to produce plasmid pCDS-Reentry-apcDNA. The pCDS-Reentry-apcDNAint2.3 construct, which contains the intron 2 and 3 of *ap* at the correct position, was synthesized by Genewiz, Inc. Transgenic flies were obtained by injecting these plasmids (300ng/μl final concentration) into *y w M{vas-int*.*Dm}zh-2A; ap* ^*attPΔCDS*^/*CyO* embryos and stocks were established according to standard genetic practice.

### Generation of *lacZ* reporter and rescue transgenic lines

To generate C1–C5 and int2.3 reporter constructs, DNA from *ap* locus was amplified by PCR from *y* ^*1*^ *w* ^*67c23*^ genomic DNA with primers containing restriction enzyme sites as overhangs, and subsequently cloned into plasmid pAttBLaZ [76] sing the respective enzymes (See S1 Table for primers and restriction enzymes). apE, apDV and apP were cloned into two reporter genes vectors, *attB*-*hs43-nuc-lacZ* [62] and *attB*-*pHPdesteGFP* [77]. The putative Pnt, Ap and Sd binding sites were identified on the basis of a bioinformatics analysis combining data from the JASPAR CORE Insecta database (http://jaspar.genereg.net/) and the Target Explorer tool [78].

Mutagenesis of the putative Pnt, Sd and Ap binding sites was performed using the QuikChange Site-Directed Mutagenesis Kit (Stratagene). See S1 Table for sequence of all primers used in this study. All the reporter constructs were inserted and analysed at the same landing attP site. The reporter FRT*-*apDV*+*E*-*FRT*-*P*-lacZ* was generated cloning PCR FRT sequences flanking the *apDV* and *apE* elements with the *apP* following the last FRT. To delete the *apDV* and *apE* casette at different time points of development we drove *flp* in the posterior compartment by crossing FRT*-*apDV*+*E*-*FRT*+*P*-lacZ* containing flies to *en*-*Gal4*, UAS-*flp*, UAS-*GFP*; *tubGal80* ^*ts*^. Larvae were kept at 17°C to keep Gal4 off. At the desired time of development, the fly vials were shifted to 29°C for *flp* induction.

*ap* rescue experiments were done replacing the *lacZ* reporter gene of the *attB*-*hs43-nuc-lacZ* with the *ap* cDNA using EcoRI and KpnI in the different *ap* CRMs combinations. All *ap* rescue transgenes were inserted in the same attP site (86Fb).

### *trx* and *Scm* mutant clonal analysis

Loss-of-function clones were generated by heat shocking the larvae for 1 hour at 37°C. The following genotypes were used:

*y w hs FLP122; FRT 82B ubiGFP*/ *FRT 82B trx* ^*E2*^

*y w hs FLP122; FRT 82B ubiGFP*/ *FRT 82B Scm* ^*D1*^

*y w hs FLP122; FRT 82B ubiGFP*/ *FRT 82B trx* ^*E2*^ *Scm* ^*D1*^

### Immunostaining

Imaginal discs were prepared and stained using standard procedures. The primary antibodies used were: rabbit and mouse anti-β-Gal (1:1000, Cappel and Promega), mouse anti-Wg (1: 50, Developmental Studies Hybridoma Bank), rat-αPS2 (1: 5, gift from Martín Bermudo) and rabbit anti-Ap (1:1000, this study)

### Chromatin immunoprecipitation experiments

Third instar larvae were dissected and wing imaginal discs were collected in PBS on ice. Discs were fixed with 1.8% formaldehyde. Chromatin preparation and immunoprecipitation were performed as described [79]. For Ap ChIPs, 1.5 μg anti-Ap (dN–20, Santa Cruz Biotechnologies) was used for each immunoprecipitation, and specificity was tested by parallel “mock” immunoprecipitations carried out with normal goat IgG (Santa Cruz Biotechnologies). ChIP enrichment values were normalized relative to “mock” enrichment values to control for any signal that could be attributed to highly accessible chromatin [80]. Three real-time PCR amplicons surrounding the apP (chr2R, 1614425–1614545; coordinates based on dm3 build of *Drosophila* genome), apE (chr2R, 1622079–1622182), or apDV (chr2R, 1639774–1639867) elements were used to quantify immunoprecipitated chromatin. For Sd ChIP, maximum enrichment signals from Sd ChIP-chip data [79] for the corresponding apP, apE, and apDV regions were normalized to the same “mock” enrichment values used in the Ap ChIP experiments. Importantly, the Sd peak at apDV was called as statistically significant in the previously published genome-wide ChIP data [79].

### Chromosome conformation capture (3C)

Chromosome conformation capture (3C) was performed as described in Webber *et al*, 2013 [81] with slight modification. Approximately 200 early third instar larvae were homogenized at room temperature in a crosslinking solution (1.8% formaldehyde, 50 mM HEPES, 1 mM EDTA, 0.5 mM EGTA, 100 mM NaCl). Total crosslinking time was limited to 20 minutes and followed by a 5-minute quench with glycine (0.125 M Glycine, 1xPBS, 0.01% Triton). Crude, fixed homogenate was then washed twice with PBS with 1% Triton, washed twice with a HEPES buffer (10 mM HEPES pH 7.6, 10 mM EDTA, 0.5 mM EGTA, 0.25% Triton), then Dounce homogenized in Buffer A (15 mM HEPES at pH 7.6, 10 mM KCl, 5 mM MgCl2, 0.1 mM EDTA, 0.5 mM EGTA, 350 mM sucrose, 1 mM DTT). After a brief centrifugation (400g for 1 minute) to remove cuticle and large debris, homogenate was centrifuged for 15 min at 10,000 rpm. Nuclei were resuspended in 100 μl of 1.2X DpnII Buffer with BSA (New England BioLabs), and then passed through a 27G syringe needle 10 times. 1.5 μl of 20% SDS was added to the nuclei-containing solution, which was then incubated for 30 min at 37°C, followed by 10 minutes at 65°C, addition of 10 μl 20% Triton X–100, and then incubation for 1 hour at 37°C. 100 units of DpnII were then added to the nuclei-containing solution, followed by overnight incubation at 37°C. The digestion reaction was stopped by adding 16 μl 10% SDS and incubating at 65°C for 10 minutes. From this point on, 3C was carried out as described in [81]. Ligation products were analyzed by qPCR (primer sequences available upon request). The amount of 3C amplicon product was normalized relative to an amplicon in the *ap* promoter that does not span a DpnII site and gives a measure of the total DNA in the reaction.

## Supporting Information

S1 Fig Wing discs and wing/notum preps of additional *ap* alleles. All 3^rd^ instar wing discs were stained for Ap (green) and Wg (red). **(A-A”)** *ap* ^*MM*^/*ap* ^*DG3*^: Ap and Wg patterns are indistinguishable from wild type. Wings look normal. This indicates that *ap* ^*MM*^ does not hamper *ap* function. **(B-D)** No Ap protein is detectable in hemizygous amorphic wing mutants *ap* ^*DG16*^, *ap* ^*DG8*^, and *ap* ^*t11b*^ (over *ap* ^*DG3*^). **(B’-D’)** Inner Wg ring is reduced to a dot, and wing pouch is lost. **(B”-D”)** No wing tissue is formed in adult flies. **(E)** *ap* ^*c2*.*73c*^/*ap* ^*DG3*^: Ap is weakly detected in the dorsal part of the wing disc (white arrow). **(E’)** Wing pouch is larger than in amophic mutants, but no D/V sub-division is observed. **(E”)** Wing stumps or small tube-like structures are often formed in adults. **(F-F”’)** *ap* ^*c1*.*60c*^/*ap* ^*DG3*^: in the weak hypomorphic mutant *ap* ^*c1*.*60c*^, *ap* is ectopically expressed in the ventral compartment correlating with the disruption of the Wg stripe at the D/V boundary (white arrow in F”’). All adult wings show notches along the wing margin (F””). All scale bars are 50 μm.
(TIF)

S2 Fig *in situ* rescue system for *ap* coding sequences. **(A)** Relative order of C2 and C5 relative to apP has no influence on wing development. Hemizygous *ap* ^*C15342*^ and *ap* ^*C52*^ over *ap* ^*DG3*^ flies develop normal wings. **(B)** Construction of *ap* ^*attPΔCDS*^: this *ap* allele harbors an attP docking site for the “coding sequence *in situ* rescue system”. Initially, attP, FRT and LoxP sites were introduced at the *ap* ^*MM*^ insertion site by direct gene conversion and ФC31-mediated recombination. This intermediate allele is referred to as *ap* ^*attBPFRTy1*^ (for details see [25]). In a second step, the complete *ap* coding sequence was deleted by *flp*-mediated recombination between the two FRT sites in *ap* ^*attBPFRTy1*^ and *ap* ^*f00878*^ and *ap* ^*attPΔCDS*^ was obtained. This deletion corresponds exactly to that in *ap* ^*DG8*^ which leads to loss of all wing and haltere structures (see Fig 1B and S1C Fig). Its attP site allows the integration of *ap* coding sequences into the endogenous *ap* locus with the help of a plasmids like pCDS-Re-entry. The offspring can be screened for transgenics thanks to the *yellow* selection marker. **(C)** At the top of the panel, allele *ap* ^*GFP*^ is shown. It contains the entire *ap* coding sequences with all introns specific for transcript *ap-RA*. The Ap protein is tagged with GFP at its C-terminal end (see [67] for a more detailed description). In *ap* ^*GFP*^ hemizygous flies, *ap* function is fully complemented. The cDNA used for the construction of *ap* ^*cDNA*^ and *ap* ^*cDNAint2*.*3*^ is also specific for transcript *ap-RA*. Introducing an intron-less cDNA is not sufficient to re-establish wild type appearing wings (*ap* ^*cDNA*^). However, it has been proposed that intron-containing genes are often transcribed more efficiently than non-intronic genes, independently of putative enhancers in intronic sequences [83]. Thus, we engineered a cDNA/gDNA hybrid containing the two short introns 2 and 3 of *ap*. The corresponding allele *ap* ^*cDNAint2*.*3*^ was obtained. Hemizygous *ap* ^*cDNAint2*.*3*^ / *ap* ^*DG3*^ flies fully rescue wing formation. **(D)** A 2.3 kb fragment containing intron 2 and 3 does not drive any detectable reporter gene expression in wing imaginal discs. Scale bars are 50 μm.
(TIF)

S3 Fig Wing-disc specific expression patterns obtained with a collection of *ap* Gal4-driver lines and reporter constructs. **(A)** A schematic representation of the *ap* genomic region is depicted by a gray bar in the center of the panel. In green, the *ap-RA* transcript is indicated along with the five conserved regions C1–C5. apP, apE and apDV correspond to the regulatory elements characterized in this study. At the top of the panel, the location of two previously reported apE containing fragments apC [24] and apRXa [25] is indicated. The horizontal bars below represent the 17 DNA elements available as Gal4 drivers (Janelia Farm database) or *lacZ*-reporter constructs. At the bottom of the panel, the wing disc specific enhancer activity of conserved regions C1 to C5 in a *lacZ* reporter assay is shown. **(B)** 4 out of 17 DNA fragments tested show activity in the dorsal wing imaginal disc. All Janelia *Gal4* lines were crossed with a stock containing UAS-*GFP* (green) and *act5C*>*stop*>*lacZ*; UAS-*flp* to lineage-trace all the cells that at one point have activated *Gal4*. Wing discs were stained for GFP (green), *lacZ* (red), Wg (blue) and Cut (red) for line 2. Note that lines 1 and 2 are active in a similar pattern in the wing pouch and hinge but are not active in the notum. Line 2 is more broadly expressed than line 1, with few cells showing activity in the ventral compartment (see arrow, Cut is in red). The other two lines active in dorsal wing disc cells are 7 and 8. They showed similar activity patterns in the notum and hinge regions with low levels in the dorsal wing pouch. Note that cells that have activated these DNA elements almost mark the entire dorsal compartment (*lacZ* in red). Also note that some cells labeled with *lacZ* of line 7 appear in the ventral compartment.
(TIF)

S4 Fig *EGFR* ^*ts*^ and *wg* experiments. **(A-D)** To reduce EGFR activity, a temperature-sensitive allele, *EGFR* ^*ts*^ was used. Larvae of the genotype *EGFR* ^*ts*^; apE*-lacZ* were maintained at 17°C and shifted to 29°C to reduce EGFR activity for a 24hr period at different time points of larval development (time interval at 29°C is indicated below each imaginal disc picture). Then larvae were returned to 17°C until dissection at around 120hrs AEL. Imaginal discs stained for apE-*lacZ* (red) and Wg (green). **(A)** Control wing imaginal disc of a larva maintained at 17°C until dissection. **(B-D)** Wing imaginal disc shifted to 29°C at mid-third **(B)**, early-third **(C)** and early-second **(D)** instar for a 24hr period. Note that apE is still active after *EGFR* removal at mid-third or early-third imaginal disc stage **(B** and **C)**. Only removal of *EGFR* function at early-second instar completely abolishes apE activity **(D)**. The resulting wing imaginal disc is strongly reduced in size and *wg* expression is lost. **(E)** *dpp*-*Gal4*; UAS-*TCF* ^*DN*^, UAS-*GFP* wing imaginal disc stained for apDV-*lacZ* (red) and GFP (green). Note that apDV activity is reduced (arrow in **E’**) although not eliminated after knockdown of the Wg pathway. Single channel is displayed for apDV-*lacZ* **(E’). (F)** Ectopic activation of the Wg pathway in *dpp*-*Gal4*; UAS-*arm* ^*S10*^, UAS-*GFP* wing imaginal disc does not ectopically activate apDV, with the exception of some scattered cells in the notum (arrow in **F’**). Single channel is displayed for apDV-*lacZ* **(F’)**. Scale bars are 50 μm.
(TIF)

S5 Fig apDV+E+P placed next to *ap*-cDNA is sufficient to rescue wing development. **(A-D)** Wing imaginal discs of different genotypes stained for *ap*-*lacZ* (green), Wg (red) and αPs2 (white, in separate channels). For each genotype, the corresponding adult wing phenotype is shown at the bottom of each panel along with details of the wing margin. *ap*-*lacZ* stands for *ap* ^*rk568*^. This is a *lacZ* enhancer trap line which behaves as a very strong *ap* allele. **(A)** *ap*-*lacZ*/+ wing imaginal discs show normal *ap-lacZ* and Wg pattern. αPS2 is restricted to ventral cells. Adult wings look normal. Dorsal and ventral patterning of the anterior wing margin is as in wild type. **(B)** *ap*-*lacZ*/*ap* ^*UGO35*^ flies are amorphic and wing imaginal discs have no wing pouch. Adult flies do not develop any wings. **(C)** *ap*-*lacZ*/*ap* ^*UGO35*^; apDV+E-*apcDNA* homozygous flies: wing imaginal discs lack the D/V Wg stripe and αPS2 is observed in the entire pouch. Wing development is partially complemented but wing margin fails to form. **(D)** *ap*-*lacZ*/*ap* ^*UGO35*^; apDV+E+P-*apcDNA* homozygous flies: in wing imaginal discs, a normal Wg D/V stripe is present and with the exception of some dorsal cells (arrow), αPS2 is restricted to ventral cells. Although wing rescue is not perfect, a clear D/V margin is observed.
(TIF)

S6 Fig Role of Trx and Scm in the regulation of *ap* expression. **(A)** Wild type wing imaginal disc stained for Wg (blue) and αPS2 (red). Note that αPS2 positive cells are confined to the ventral compartment. **(B)** *trx* ^*E2*^ mutant clones generated 48–72hrs AEL: clones are marked by the absence of GFP. Discs were stained for Wg (blue) and αPS2 (red). **(B’)** single-channel picture of **(B)**: *αPS2* is derepressed in dorsal *trx* ^*E2*^ clones (green arrow). **(C)** *ap-lacZ* (*ap* ^*rK568*^) expression in *Scm* ^*D1*^ clones generated 48–72hrs AEL: clones are marked by the absence of GFP (several outlined in white). Discs were stained for Wg (blue) and *ap*-*lacZ* (red). **(C’)** *ap*-*lacZ* expression is derepressed in ventral clones close to the D/V boundary. **(C”-C”’)** Close-up of **(C)**. Note *ap-lacZ* derepression in *Scm* ^*D1*^mutant cells close to the D/V **(C”)**. *wg* expression does not follow *ap-lacZ* derepression **(C”’)**. **(D)** *ap-LacZ* expression in *Scm* ^*D1*^ *trx* ^*E2*^ double mutant clones generated 48–72hrs AEL: clones are marked by the absence of GFP (several outlined in white). Discs were stained for Wg (blue) and for *ap*-*lacZ* (red). **(D’)** *ap*-*lacZ* expression is downregulated in dorsal cells but no derepression is observed in ventral cells. **(D”-D”’)** Close-up of (D’). Note *ap-lacZ* downregulation in the dorsal compartment in *Scm* ^*D1*^ *trx* ^*E2*^ mutant cells **(D”)**. *wg* expression is not altered in *Scm* ^*D1*^ *trx* ^*E2*^ mutant cells. In particular, *wg* is not ectopically expressed along the edge of clones with reduced *ap-lacZ* activity **(D”’)**. D, dorsal and V, ventral.
(TIF)

S7 Fig apP, apE and apDV cooperate best when *in cis*. **(A)** In hemizygous +/*ap* ^*DG3*^ flies, *ap* and *wg* expression patterns in wing discs are normal **(A’, A”)**. Apart from rare, mild margin defects, most wings are indistinguishable from wild type **(A”’)**. Note that the 3 *ap* CRMs are all *in cis*. **(B)** *ap* ^*DG1*^/*ap* ^*t11b*^: apP is on one chromosome and apE and apDV are on the other. *ap* ^*DG1*^ and *ap* ^*t11b*^ alleles are amorphic when tested in hemizygous condition. *In trans* to each other, wing development is much improved. Typically, <20% of the wings appear normal. Among the rest, wings displaying an enlarged posterior compartment are frequent **(B”’)**. Wing margin is rather well formed. Consistent with the adult phenotype, and although *ap* expression appears fairly normal, the posterior compartment is often overgrown in imaginal wing discs and the Wg stripe along the D/V border is wavy. **(B’ and B”)**. **(C)** *ap* ^*DG14*^/*ap* ^*DG12*^: formally, this genotype is equivalent to *ap* ^*C1345*^/*ap* ^*C1234*^ shown in Fig 7A. apE and apDV are present *in trans* to each other. **(C’ and C”)** Expression of *ap* is affected in the dorsal compartment, leading to *wg* misexpression. **(C”’)** All adult wings have similar phenotypes, including large, unstructured outgrowths. **(D)** *ap* ^*f00451*^/*ap* ^*DG3*^: on *ap* ^*f00451*^, apE and apDV enhancers are separated by a cluster of Su(Hw) binding sites. Many studies have shown that such clusters interfere with enhancer-promoter communication. **(D’-D”’)** The phenotypes observed in all *ap* ^*f00451*^/ *ap* ^*DG3*^ discs and adult wings suggest that apDV is not completely excluded from *ap* regulation. Their appearances are similar to those observed for *ap* ^*DG14*^/*ap* ^*DG12*^ animals. From the similarities of the phenotypes, it may be inferred that *in trans* configuration of apE and apDV is equivalent to partially blocking apDV from interaction with apP. Scale bars are 50 μm.
(TIF)

S1 Table Primer sequences used in this study. Primers used for the cloning of the different CRMs (the respective restriction enzymes used for cloning are indicated in the primer names). Mutagenesis of the Pnt, Sd and Ap putative binding sites (in bold) was performed using the QuikChange Site-Directed Mutagenesis Kit (Stratagene).
(TIF)
