# Supplementary figures and images for: Establishment of a Developmental Compartment Requires Interactions between Three Synergistic Cis-regulatory Modules
Source: PLoS Genet. 2015 Oct 15;11(10):e1005376. doi: 10.1371/journal.pgen.1005376 (PMC4607503; doi:10.1371/journal.pgen.1005376)

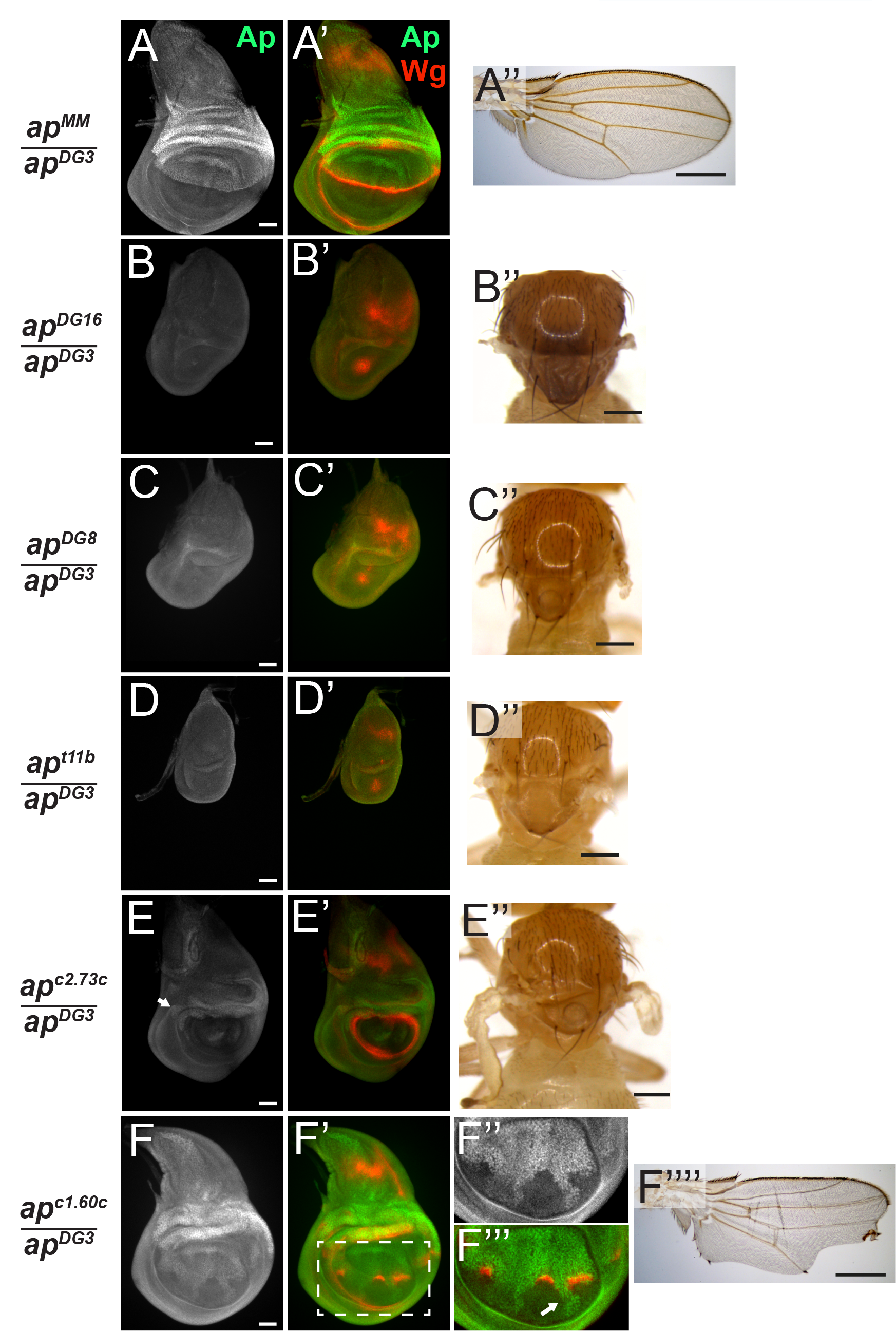

Supplement: S1 Fig — All 3rd instar wing discs were stained for Ap (green) and Wg (red). (A-A”) ap MM/ap DG3: Ap and Wg patterns are indistinguishable from wild type. Wings look normal. This indicates that ap MM does not hamper ap function. (B-D) No Ap protein is detectable in hemizygous amorphic wing mutants ap DG16, ap DG8, and ap t11b (over ap DG3). (B’-D’) Inner Wg ring is reduced to a dot, and wing pouch is lost. (B”-D”) No wing tissue is formed in adult flies. (E) ap c2.73c/ap DG3: Ap is weakly detected in the dorsal part of the wing disc (white arrow). (E’) Wing pouch is larger than in amophic mutants, but no D/V sub-division is observed. (E”) Wing stumps or small tube-like structures are often formed in adults. (F-F”’) ap c1.60c/ap DG3: in the weak hypomorphic mutant ap c1.60c, ap is ectopically expressed in the ventral compartment correlating with the disruption of the Wg stripe at the D/V boundary (white arrow in F”’). All adult wings show notches along the wing margin (F””). All scale bars are 50 μm. (TIF) [file pgen.1005376.s001.tif]

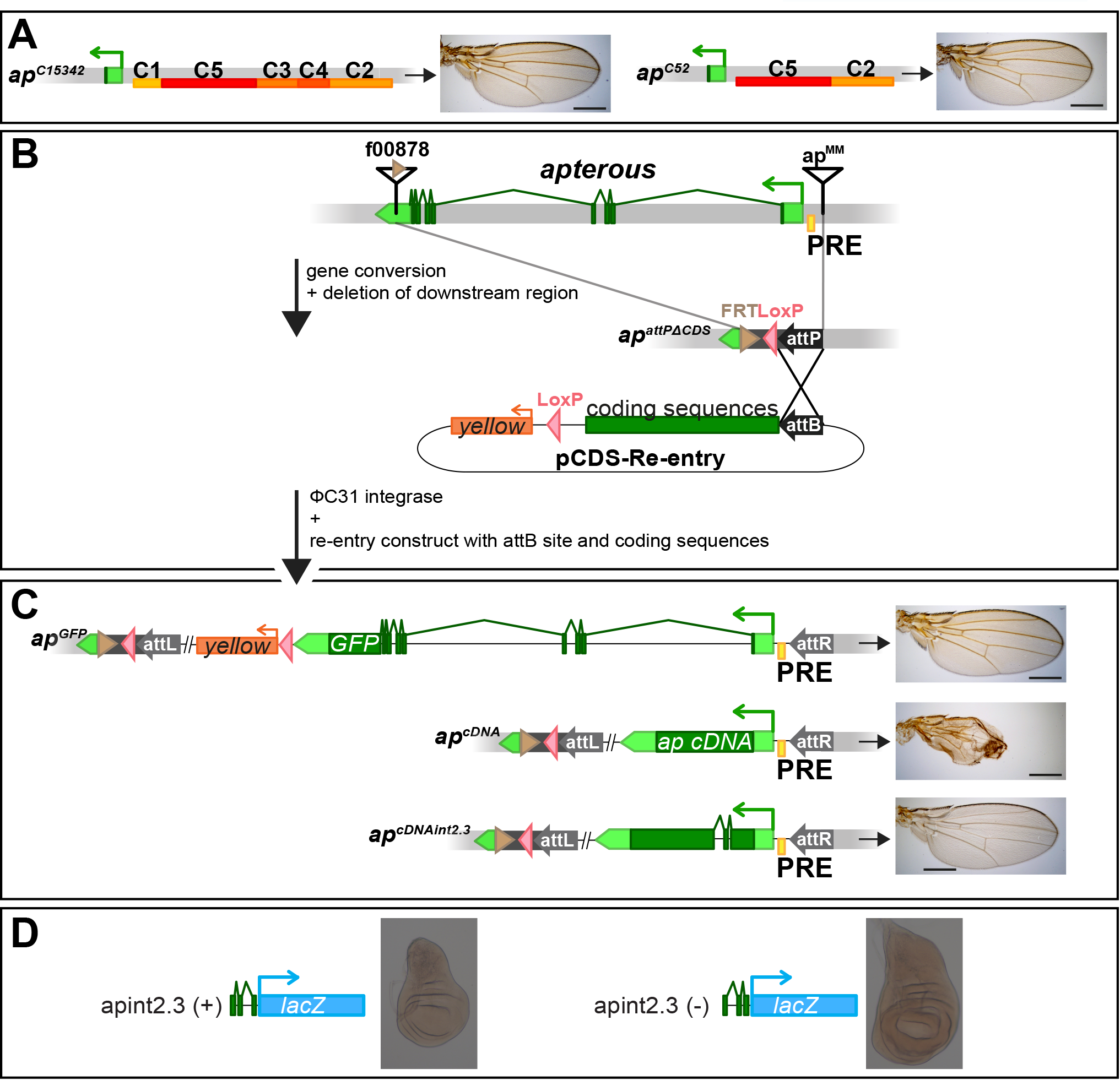

Supplement: S2 Fig — (A) Relative order of C2 and C5 relative to apP has no influence on wing development. Hemizygous ap C15342 and ap C52 over ap DG3 flies develop normal wings. (B) Construction of ap attPΔCDS: this ap allele harbors an attP docking site for the “coding sequence in situ rescue system”. Initially, attP, FRT and LoxP sites were introduced at the ap MM insertion site by direct gene conversion and ФC31-mediated recombination. This intermediate allele is referred to as ap attBPFRTy1 (for details see [25]). In a second step, the complete ap coding sequence was deleted by flp-mediated recombination between the two FRT sites in ap attBPFRTy1 and ap f00878 and ap attPΔCDS was obtained. This deletion corresponds exactly to that in ap DG8 which leads to loss of all wing and haltere structures (see Fig 1B and S1C Fig). Its attP site allows the integration of ap coding sequences into the endogenous ap locus with the help of a plasmids like pCDS-Re-entry. The offspring can be screened for transgenics thanks to the yellow selection marker. (C) At the top of the panel, allele ap GFP is shown. It contains the entire ap coding sequences with all introns specific for transcript ap-RA. The Ap protein is tagged with GFP at its C-terminal end (see [67] for a more detailed description). In ap GFP hemizygous flies, ap function is fully complemented. The cDNA used for the construction of ap cDNA and ap cDNAint2.3 is also specific for transcript ap-RA. Introducing an intron-less cDNA is not sufficient to re-establish wild type appearing wings (ap cDNA). However, it has been proposed that intron-containing genes are often transcribed more efficiently than non-intronic genes, independently of putative enhancers in intronic sequences [83]. Thus, we engineered a cDNA/gDNA hybrid containing the two short introns 2 and 3 of ap. The corresponding allele ap cDNAint2.3 was obtained. Hemizygous ap cDNAint2.3 / ap DG3 flies fully rescue wing formation. (D) A 2.3 kb fragment containing intron 2 and 3 does [file pgen.1005376.s002.tif]

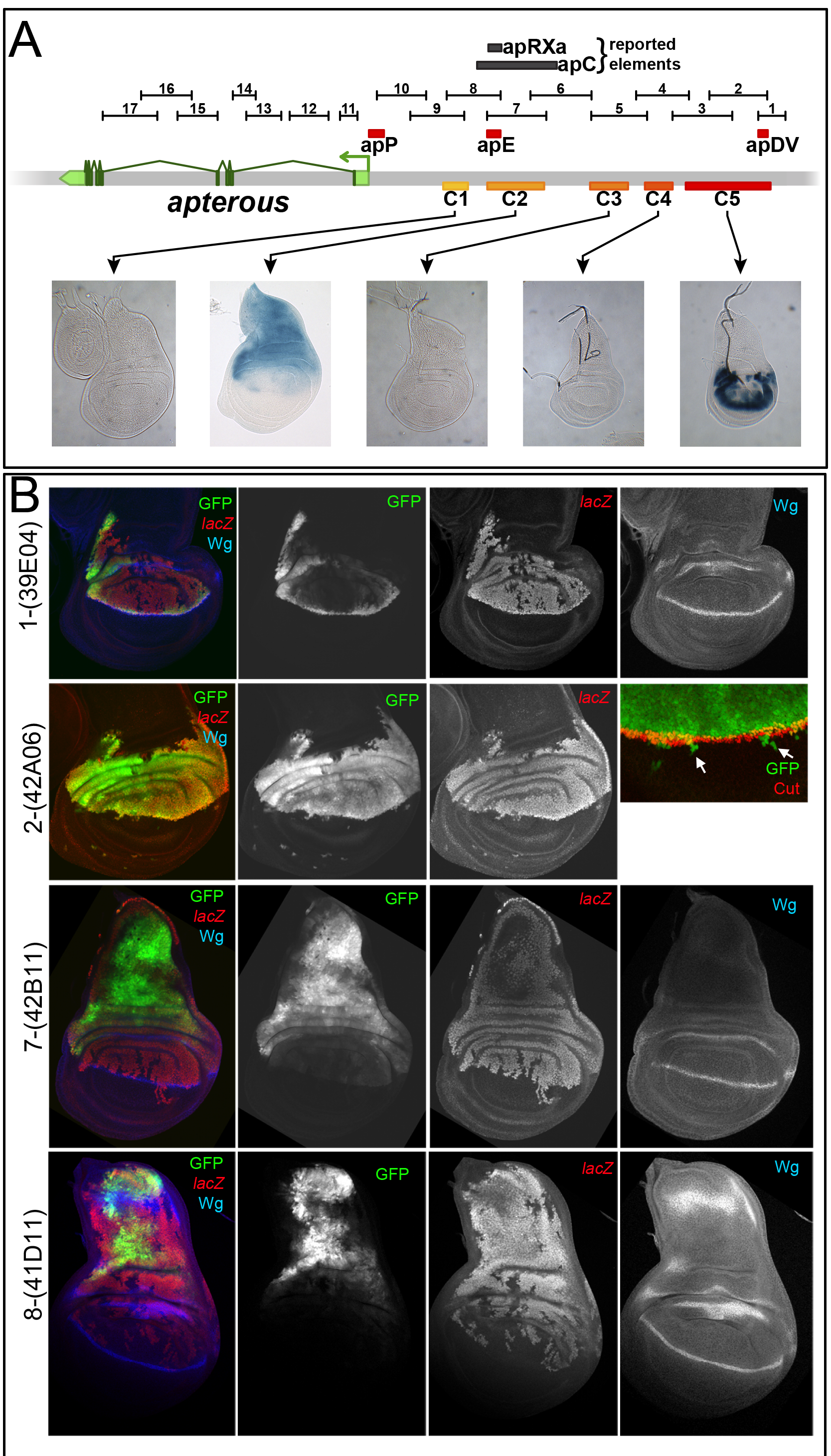

Supplement: S3 Fig — (A) A schematic representation of the ap genomic region is depicted by a gray bar in the center of the panel. In green, the ap-RA transcript is indicated along with the five conserved regions C1–C5. apP, apE and apDV correspond to the regulatory elements characterized in this study. At the top of the panel, the location of two previously reported apE containing fragments apC [24] and apRXa [25] is indicated. The horizontal bars below represent the 17 DNA elements available as Gal4 drivers (Janelia Farm database) or lacZ-reporter constructs. At the bottom of the panel, the wing disc specific enhancer activity of conserved regions C1 to C5 in a lacZ reporter assay is shown. (B) 4 out of 17 DNA fragments tested show activity in the dorsal wing imaginal disc. All Janelia Gal4 lines were crossed with a stock containing UAS-GFP (green) and act5C>stop>lacZ; UAS-flp to lineage-trace all the cells that at one point have activated Gal4. Wing discs were stained for GFP (green), lacZ (red), Wg (blue) and Cut (red) for line 2. Note that lines 1 and 2 are active in a similar pattern in the wing pouch and hinge but are not active in the notum. Line 2 is more broadly expressed than line 1, with few cells showing activity in the ventral compartment (see arrow, Cut is in red). The other two lines active in dorsal wing disc cells are 7 and 8. They showed similar activity patterns in the notum and hinge regions with low levels in the dorsal wing pouch. Note that cells that have activated these DNA elements almost mark the entire dorsal compartment (lacZ in red). Also note that some cells labeled with lacZ of line 7 appear in the ventral compartment. (TIF) [file pgen.1005376.s003.tif]

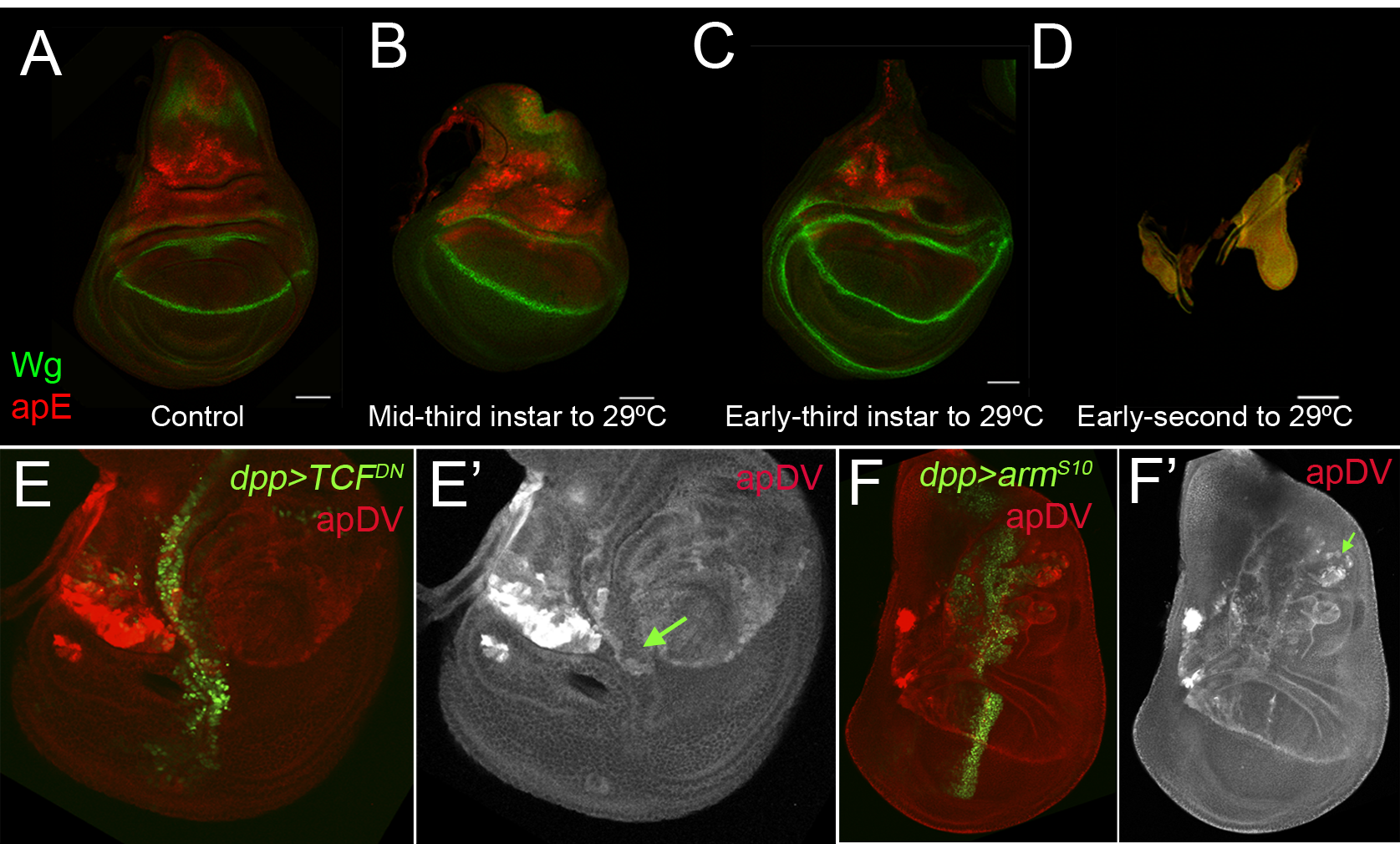

Supplement: S4 Fig — (A-D) To reduce EGFR activity, a temperature-sensitive allele, EGFR ts was used. Larvae of the genotype EGFR ts; apE-lacZ were maintained at 17°C and shifted to 29°C to reduce EGFR activity for a 24hr period at different time points of larval development (time interval at 29°C is indicated below each imaginal disc picture). Then larvae were returned to 17°C until dissection at around 120hrs AEL. Imaginal discs stained for apE-lacZ (red) and Wg (green). (A) Control wing imaginal disc of a larva maintained at 17°C until dissection. (B-D) Wing imaginal disc shifted to 29°C at mid-third (B), early-third (C) and early-second (D) instar for a 24hr period. Note that apE is still active after EGFR removal at mid-third or early-third imaginal disc stage (B and C). Only removal of EGFR function at early-second instar completely abolishes apE activity (D). The resulting wing imaginal disc is strongly reduced in size and wg expression is lost. (E) dpp-Gal4; UAS-TCF DN, UAS-GFP wing imaginal disc stained for apDV-lacZ (red) and GFP (green). Note that apDV activity is reduced (arrow in E’) although not eliminated after knockdown of the Wg pathway. Single channel is displayed for apDV-lacZ (E’). (F) Ectopic activation of the Wg pathway in dpp-Gal4; UAS-arm S10, UAS-GFP wing imaginal disc does not ectopically activate apDV, with the exception of some scattered cells in the notum (arrow in F’). Single channel is displayed for apDV-lacZ (F’). Scale bars are 50 μm. (TIF) [file pgen.1005376.s004.tif]

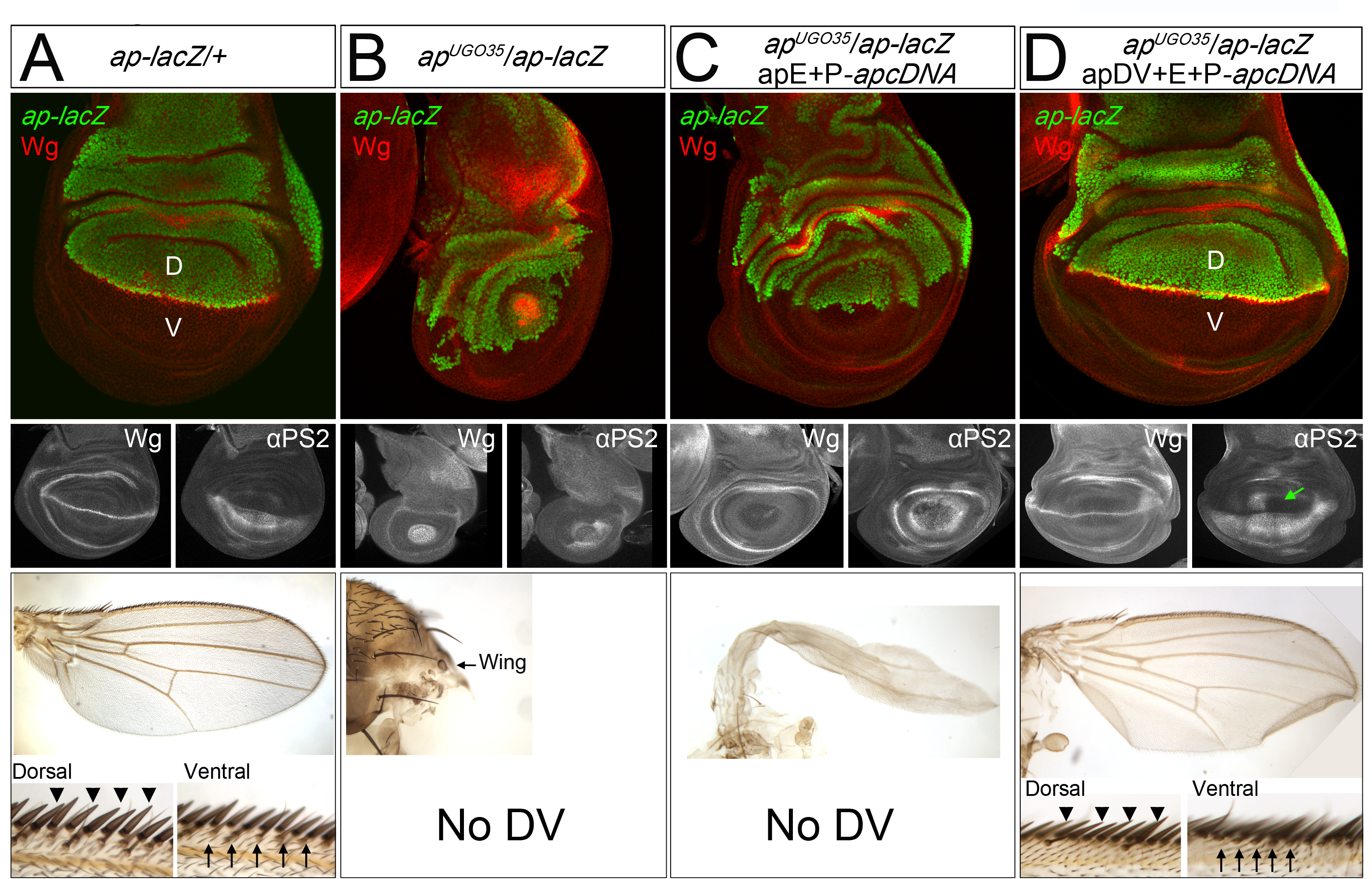

Supplement: S5 Fig — (A-D) Wing imaginal discs of different genotypes stained for ap-lacZ (green), Wg (red) and αPs2 (white, in separate channels). For each genotype, the corresponding adult wing phenotype is shown at the bottom of each panel along with details of the wing margin. ap-lacZ stands for ap rk568. This is a lacZ enhancer trap line which behaves as a very strong ap allele. (A) ap-lacZ/+ wing imaginal discs show normal ap-lacZ and Wg pattern. αPS2 is restricted to ventral cells. Adult wings look normal. Dorsal and ventral patterning of the anterior wing margin is as in wild type. (B) ap-lacZ/ap UGO35 flies are amorphic and wing imaginal discs have no wing pouch. Adult flies do not develop any wings. (C) ap-lacZ/ap UGO35; apDV+E-apcDNA homozygous flies: wing imaginal discs lack the D/V Wg stripe and αPS2 is observed in the entire pouch. Wing development is partially complemented but wing margin fails to form. (D) ap-lacZ/ap UGO35; apDV+E+P-apcDNA homozygous flies: in wing imaginal discs, a normal Wg D/V stripe is present and with the exception of some dorsal cells (arrow), αPS2 is restricted to ventral cells. Although wing rescue is not perfect, a clear D/V margin is observed. (TIF) [file pgen.1005376.s005.tif]

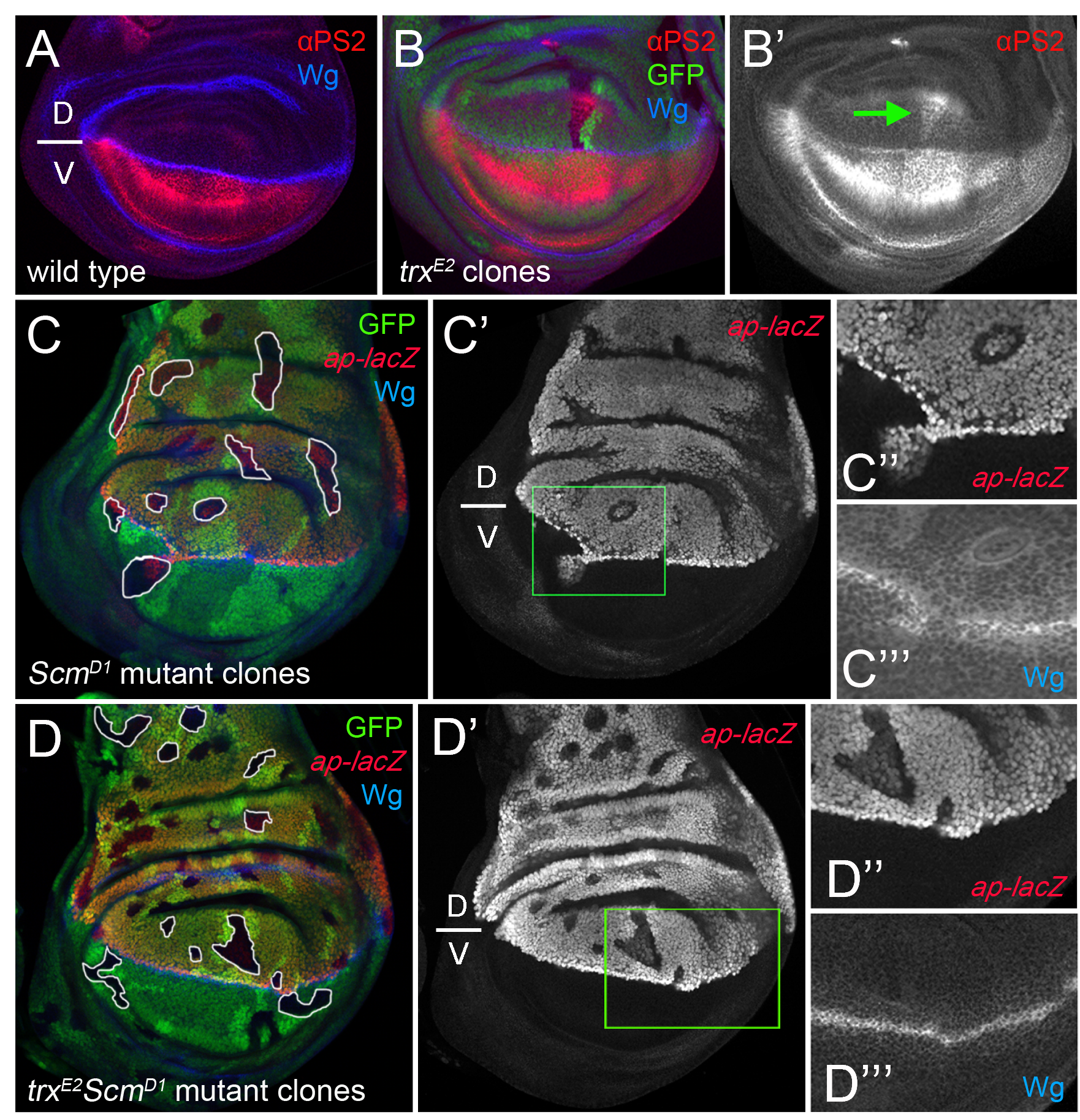

Supplement: S6 Fig — (A) Wild type wing imaginal disc stained for Wg (blue) and αPS2 (red). Note that αPS2 positive cells are confined to the ventral compartment. (B) trx E2 mutant clones generated 48–72hrs AEL: clones are marked by the absence of GFP. Discs were stained for Wg (blue) and αPS2 (red). (B’) single-channel picture of (B): αPS2 is derepressed in dorsal trx E2 clones (green arrow). (C) ap-lacZ (ap rK568) expression in Scm D1 clones generated 48–72hrs AEL: clones are marked by the absence of GFP (several outlined in white). Discs were stained for Wg (blue) and ap-lacZ (red). (C’) ap-lacZ expression is derepressed in ventral clones close to the D/V boundary. (C”-C”’) Close-up of (C). Note ap-lacZ derepression in Scm D1mutant cells close to the D/V (C”). wg expression does not follow ap-lacZ derepression (C”’). (D) ap-LacZ expression in Scm D1 trx E2 double mutant clones generated 48–72hrs AEL: clones are marked by the absence of GFP (several outlined in white). Discs were stained for Wg (blue) and for ap-lacZ (red). (D’) ap-lacZ expression is downregulated in dorsal cells but no derepression is observed in ventral cells. (D”-D”’) Close-up of (D’). Note ap-lacZ downregulation in the dorsal compartment in Scm D1 trx E2 mutant cells (D”). wg expression is not altered in Scm D1 trx E2 mutant cells. In particular, wg is not ectopically expressed along the edge of clones with reduced ap-lacZ activity (D”’). D, dorsal and V, ventral. (TIF) [file pgen.1005376.s006.tif]

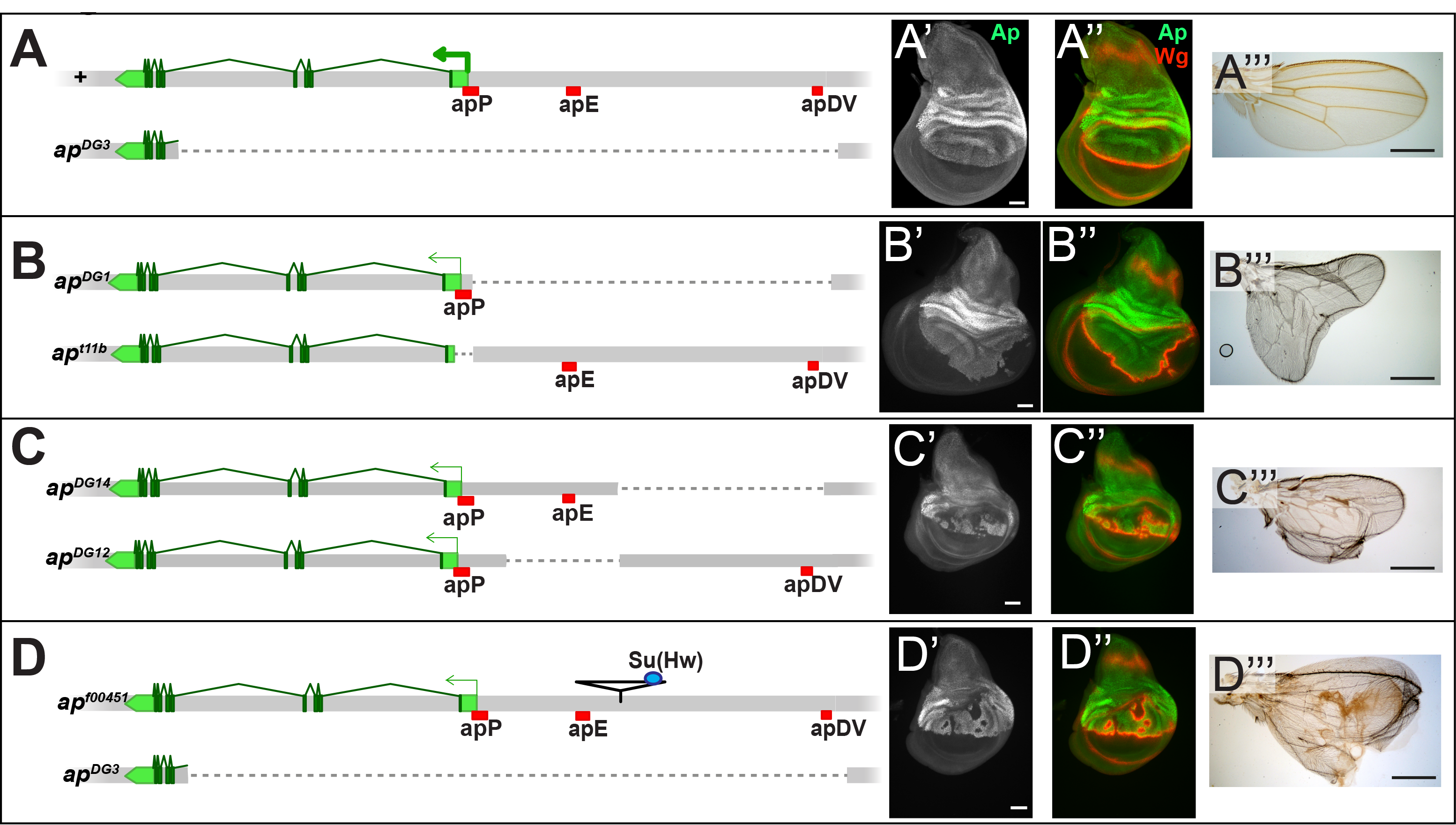

Supplement: S7 Fig — (A) In hemizygous +/ap DG3 flies, ap and wg expression patterns in wing discs are normal (A’, A”). Apart from rare, mild margin defects, most wings are indistinguishable from wild type (A”’). Note that the 3 ap CRMs are all in cis. (B) ap DG1/ap t11b: apP is on one chromosome and apE and apDV are on the other. ap DG1 and ap t11b alleles are amorphic when tested in hemizygous condition. In trans to each other, wing development is much improved. Typically, <20% of the wings appear normal. Among the rest, wings displaying an enlarged posterior compartment are frequent (B”’). Wing margin is rather well formed. Consistent with the adult phenotype, and although ap expression appears fairly normal, the posterior compartment is often overgrown in imaginal wing discs and the Wg stripe along the D/V border is wavy. (B’ and B”). (C) ap DG14/ap DG12: formally, this genotype is equivalent to ap C1345/ap C1234 shown in Fig 7A. apE and apDV are present in trans to each other. (C’ and C”) Expression of ap is affected in the dorsal compartment, leading to wg misexpression. (C”’) All adult wings have similar phenotypes, including large, unstructured outgrowths. (D) ap f00451/ap DG3: on ap f00451, apE and apDV enhancers are separated by a cluster of Su(Hw) binding sites. Many studies have shown that such clusters interfere with enhancer-promoter communication. (D’-D”’) The phenotypes observed in all ap f00451/ ap DG3 discs and adult wings suggest that apDV is not completely excluded from ap regulation. Their appearances are similar to those observed for ap DG14/ap DG12 animals. From the similarities of the phenotypes, it may be inferred that in trans configuration of apE and apDV is equivalent to partially blocking apDV from interaction with apP. Scale bars are 50 μm. (TIF) [file pgen.1005376.s007.tif]

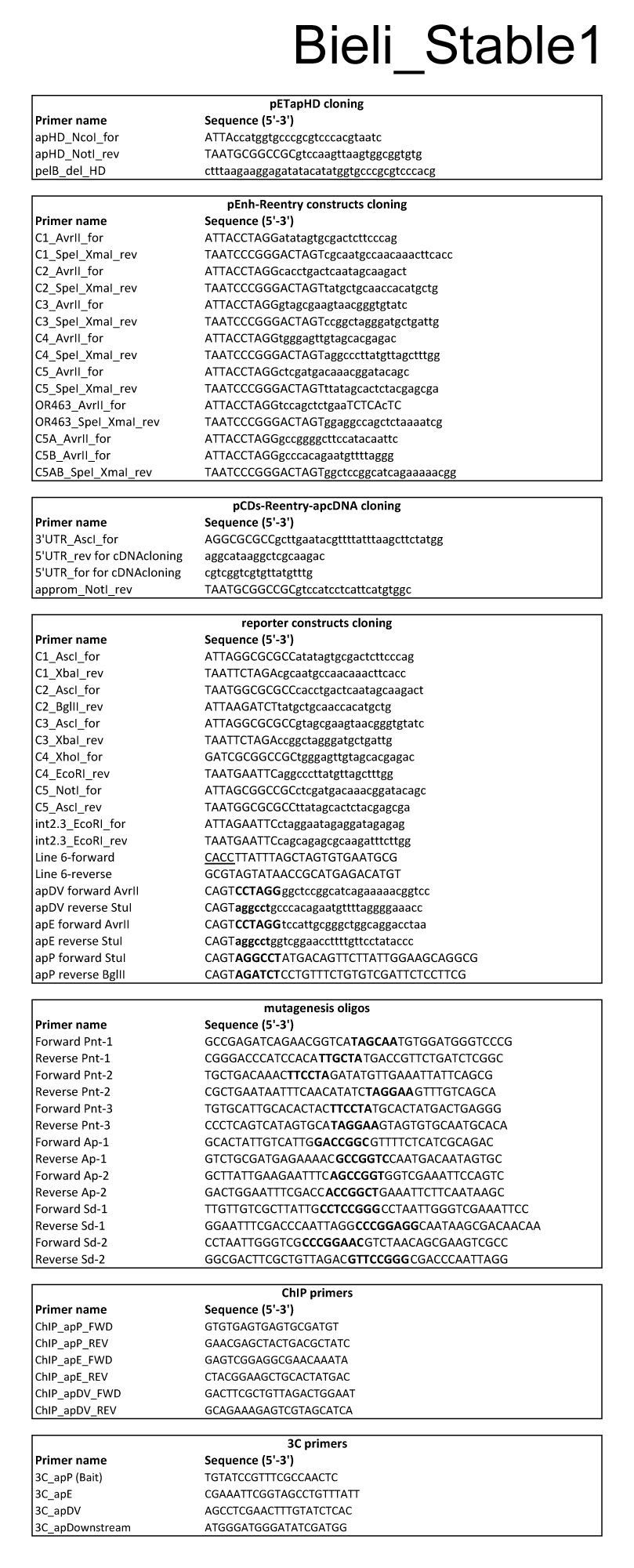

Supplement: S1 Table — Primers used for the cloning of the different CRMs (the respective restriction enzymes used for cloning are indicated in the primer names). Mutagenesis of the Pnt, Sd and Ap putative binding sites (in bold) was performed using the QuikChange Site-Directed Mutagenesis Kit (Stratagene). (TIF) [file pgen.1005376.s008.tif]
